# Teaching data science to undergraduate translation trainees: Pilot evaluation of a task-based course

**DOI:** 10.3389/fpsyg.2022.939689

**Published:** 2022-08-03

**Authors:** Da Yan, Junyue Wang

**Affiliations:** School of Foreign Languages, Xinyang Agriculture and Forestry University, Xinyang, China

**Keywords:** translation technology, natural language processing, data science, undergraduate translation training, pilot evaluation

## Abstract

The advancement in technology has changed the workflow and the role of human translator in recent years. The impact from the trend of technology-mediated translation prompted the ratification of technology literacy as a major competence for modern translators. Consequently, teaching of translation technology including but not limited to Computer-aided Translation (CAT) and Machine Translation (MT) became part of comprehensive curricula for translation training programs. However, in many institutions, the teaching of translation technology was haunted by issues such as: narrow scope of curriculum design, outdated technologies, and unbalance between theories and practices in teaching. The study was the pilot evaluation of a tailored course to foster translation trainees’ knowledge and abilities of data science. The course was designed to be a fundamental step toward sophisticated translation technologies. During the pilot evaluation of the 8-week course, 85 students (*n* = 85) were recruited as participants. The study adopted a mix-method design by employing a survey to investigate student’s level of satisfaction toward the course and focus group discussion to understand students’ attitudes and perceptions of key aspects of the course. By interpreting the results from statistical analysis of the survey (5.39/7) and thematic analysis of the focus group discussion, the course of data science for translators was well received among participants. The evaluation project manifested the feasibility and effectiveness of a translator-oriented data science course.

## Introduction

### Background

Propelled by recent advancement in Information and Communication Technology (ICT), Natural Language Processing (NLP) and Machine Learning (ML), dramatic changes have taken place in translation and translation education. Take Computer-aided Translation (CAT) and Machine Translation (MT) for example, after initial distrust in their affordance to enhance translators’ performance in professional settings (Wu et al., 2012; Koehn, 2015; Sam et al., 2015; Bundgaard et al., 2016; Sun, 2021), these technologies have become tools of trade for professionals in recent years (Olohan, 2011; Cadwell et al., 2018; Vieira, 2020; Vieira et al., 2021). After generations of technological upgrades, new technologies have been closely integrated with a variety of translation domains: from audiovisual translation (Toral et al., 2018), clinical medicine (Khoong and Rodriguez, 2022), business activities (Bowker, 2020) to tourism (Narzary et al., 2019).

The impact of technology in language service industry resulted in the flourishing development of translation technology education for translation trainees (Man et al., 2020). A growing number of technology courses were developed and taught across the globe (Toral et al., 2018; Su, 2021). The prosperity in translation technology education consolidated the importance of technology literacy in translation training. In many programs and translation competence frameworks, digital literacy and information literacy have become an indispensable part of the curriculum (Ivanova, 2016; Melby and Hague, 2019). For example, in comparison of the 2003 and 2017 editions of PACTE’s translation competence model, digital literacy has become detailly documented and emphasized as a subcategory of instrumental sub-competence (Albir et al., 2020).

Since 2018, China’s Ministry of Education advocated the construction of “New Liberal Art” in Chinese universities to bring about reform in undergraduate education with Chinese characteristics (Wang and Tian, 2019). According to Li et al. (2020), the shift toward “New Liberal Art” in an era of artificial intelligence called for technology-oriented changes of undergraduate to embrace the stage-of-the-art technologies. The proposal to incorporate technology education in the curriculum of arts was in tandem with the argument by Seldon and Adiboye (2018) that “data literacy” and “technology literacy” should be included as the objectives of higher education. Wu (2019 p. 6), director of Higher Education Department, Ministry of Education, suggested that development of foreign language education in China should “not resist or despise technology but emphasize and welcome” new breakthroughs in technology.

However, curriculum reform and development of new courses to enhance translation students’ technology literacy within the context of “New Liberal Art” were rare to be found. Existing studies focused on the reform in curriculum and educational paradigm from a theoretical perspective. For example, in a paper on educational changes for translation technology education (Huang, 2021), a set of pedagogical reform policies were proposed without reporting prior empirical experience. To truly connect the development of BTI education with the demands from job market, and incorporate new technologies in translation education, changes should be made to translation education curriculum development for the “changes of tomorrow” (Yu and Dong, 2021, 84).

### Review of literature

#### Translation technology education

In line with the flourishing development of CAT and MT, the impact of technology ushered in the development of translation technology education in various forms (Alcina et al., 2007; Erwen and Wenming, 2013; Alkan, 2016; Jiménez-Crespo, 2017; Wu, 2021). At the beginning, the teaching of translation technology started as discrete courses within the training of translators, but the significance and applicability of translation technology gradually evoked the inclusion of translation technology abilities as one of the core objectives of translation training (Doherty and Kenny, 2014; Gaspari et al., 2015; Mellinger, 2017). With the consolidation of the position of translation technology as a pivotal competence for translators, growing numbers of interdisciplinary courses were developed and taught (O’Brien and Ehrensberger-Dow, 2020; Mikhailov, 2021; Krüger, 2021a,b). In addition to course development, upgrades in learning environment and innovation in learning approaches took place. Translation technology laboratories specially designed for the needs of translation education was built (Doherty and Moorkens, 2013). Efforts were made to enhance the learning of translation technology with alternative learning, such as ePortfolio (Rico, 2017), project-based learning approach (Mitchell-Schuitevoerder, 2020), and lifelong learning (Enriquez Raido, 2013).

In the context of Chinese translation education, translation technology courses has been widely developed and taught in BTI programs in many universities in recent years (Wang and Liu, 2022). For its wide application in modern language service industry, CAT became the *de facto* standard of translation technology to be taught at undergraduate level in China (Erwen and Wenming, 2013). Advanced translation technology such as MT was also taught in BTI programs, but less frequently (Yang and Mustafa, 2022). According to the statistics of a survey on translation technology education in China, 55.8% of the universities with accredited translation programs had technology courses, but the teaching of CAT accounted for 90.4% of all available courses (Wang et al., 2018). Under the facade of exuberance, underlying issues as relatively low quality of existing curriculum, dated instructional methods, and underestimation of the value of translation technology were unaddressed (Wang, 2012; Wang et al., 2018; Zhang et al., 2021). Comparing the realities of translation technology education in China with the requirements from national standards, Huang (2021, 82) warned that “providing only CAT courses was far from enough to cultivate students with adequate translation technology literacy.”

#### Teaching programming to translation trainees

In recent years, innovation has been made in translation technology education to enhance translation learners’ ability in programming.

In a MT course, the lecturer used an online repository of python resources for students to run common machine translation tasks as “exploring word embeddings, preparing MT training data, training … machine translation systems or calculating automatic MT quality metrics” (Krüger, 2021a, 4). Krüger (2021a,b p. 16) argued that programming skills were not a must for students to use the repository of codes since learners with no programming abilities could “focus on the actual domain knowledge” while those programming-savvy could use the resources interactively.

Since September 2015, pilot courses were developed to teach relevant knowledge in “Computer Basics, Website Development, Database Principle, Translation Technologies, Natural Language Processing” for 80 students from a BTI program in China (Han, 2019). In regards to programming teaching, the official course included introductory Python programming contents and intermediate knowledge of using Python for processing multilingual documents, and web application development (Han and Liu, 2020).

However, the attempts to embed teaching and learning of programming languages in a translation trainee program were still rare, in China and the globe. Being the first reported case in incorporating programming skills as part of the curriculum for translation training, the evaluation and learning outcomes of the pilot courses pointed to a direction that should be followed for innovation in translation education. According to the BTI program decision-makers Han and Liu (2020, 62) at Beijing Language and Culture University, apart from success in their initial innovation in cultivation undergraduate talents from an interdisciplinary perspective, efforts should be made to polish the curriculum in the following-up stages of program development. In China, with nearly 300 colleges and universities providing accredited translation training programs across the nation (Tao, 2019), the question of “how to teach technology” and “what to teach technology” needed to be answered (Yu, 2021).

The reforms and innovations to translation technology education, as part of the shift toward “New Liberal Art” in China’s undergraduate education, would be significant for educators, researchers and stakeholders from the international community as well. In the past, Pym’s Minimalist Approach in defining translation competence (Pym, 2003) contributed to the favorable stance toward minimalistic approach of translation technology education against the maximalist approach (Austermuehl, 2013). Maximalist approach received criticism for its focus on “the temporary and unstable needs of the industry” (Çeti̇ner, 2021, 251). Nevertheless, in an era when translation technology was widely used and the evaluation of translation competence has changed, would the criticism the still stand? If so, the experiences from reform and innovation in the Chinese context would be valuable to a broader audience.

### Research objectives

Against the backdrop of new trends in development of undergraduate translation technology education in China, researchers of the present study tried to develop a new course to teach basic data science for undergraduate translation trainees. The primary objective of the study was to investigate participants’ level of satisfaction toward the course. Additionally, respondent’ attitude and perception about the course were collected and reviewed.

## Context and motivation

### Context

The research took place in an undergraduate university in China (whose name is omitted for anonymity and hereinafter abbreviated as *the university*). The Bachelor of Translation and Interpreting (BTI) program at *the university* was established since 2018. By April 2022, there were ~450 trainees receiving translation training at the institution as BTI degree candidates.

**Figure 1:**
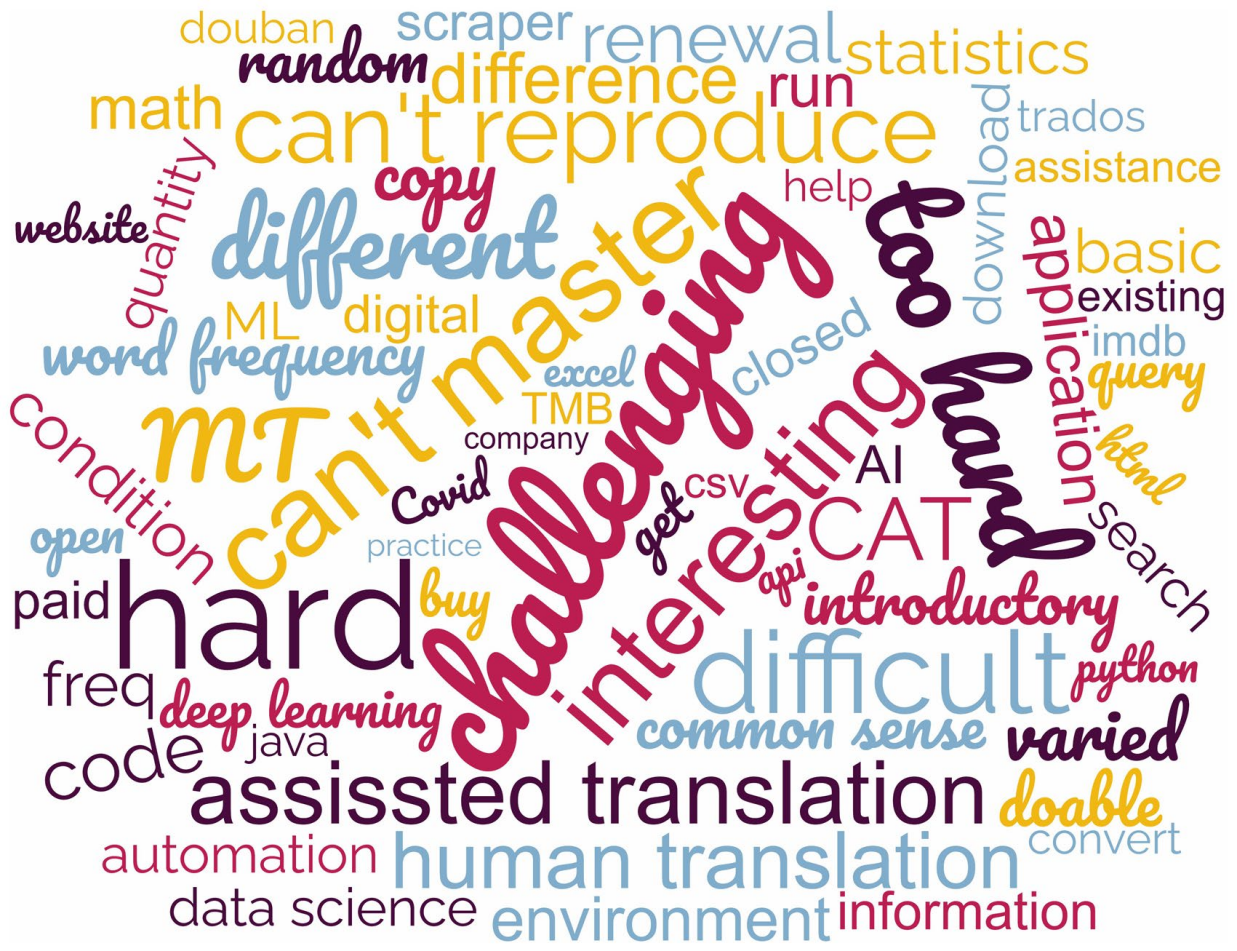
Word cloud of students’ feedback.

Translation technology was taught in the university in the form of “one dedicated course plus one training workshop” mode. Specifically, the lecture-based course was entitled “Computer-aided Translation” and the workshop was a 30-h condensed session of modern translation technologies carried out within 1 week. Based on the students’ feedback, learning outcomes and self-efficacy of students toward translation technology literacy and abilities were not satisfactory. The most common complaints extracted from the feedback were “interesting but challenging,” “I cannot reproduce on my own after class,” “I can only finish the task with data and documents provided by the teacher, but I do not know how to create my own” and “I wish to learn some basics instead of how to use the software.” Figure 1 shows the translated version of word cloud graph made with an online word cloud generator (Zygomatic, 2003) with data extracted from students’ feedback. The size of words in the word cloud graph represented their frequency from the feedback.

### Motivation

Based on the observed challenges and issues, the BTI program director called for development of new translation technology courses. The course of the present study was an introductory course to teach basic data science skills, with condescend session of CAT and corpus as components of the course. The revamping of the existing courses was also supported by school and university administrators. Since the academic year of 2021, the development of the new course kicked off, and the first pilot implementation and evaluation were carried out since the academic year 2022.

### Course description

The course of data science entitled *“Introductory data science for translators”* was designed to cultivate students’ ability to understand, wrangle, transform and manipulate text data across the internet. Being an introductory course for undergraduate students, the course was interdisciplinary by connecting various fields related to translation technology: data mining, natural language processing, machine translation, etc. Students were expected to learn basic knowledge of data science and its application in translation. Student were expected to apply knowledge acquired from the course in other translation technology courses such as CAT for better performance and insightful understanding.

The course followed a task-based design. Task-based learning proved effective in language teaching and learning, when supported by administers and practiced properly (Subekti, 2020; Xu and Fan, 2022). The course was composed of eight different tasks with incremental difficulties (see Table 1 for course specifications). Sample source code and reading materials were provided to students in order to facilitate self-learning after classroom instruction. Students were required to submit their solution for each task within 2 weeks. Throughout the course, self-directed out-of-class learning is accentuated. All instruction materials and supplementary materials are provided to students in PDF document format (see Supplementary Appendix 1 for sample instructional material which is a tutorial for the construction of a subtitle corpus).

**Table 1:** Course specifications.

No.	Week	Task	Contents
**1**	Week 1 and Week 2	Structured Text Editing	Understanding markup language Learning to write and edit html Building static web pages From text to csv.
**2**	Week 3 and Week 4	Python (I)	Learning Python basics Code to generate html Use NLTK package to analyze text Using Pandas to analyze numeric data.
**3**	Week 5 and Week 6	Python (II)	Web scraping with requests and lxml Scraping COVID-19 data from news websites into spreadsheets or csv files Analyze COVID-19 data Data visualization of COVID-19 data.
**4**	Week 7 and Week 8	API and Translation	Using API for translation automation Making your own API with Flask Using API to provide auto translation service.
**5**	Week 9 and Week 10	Corpus Linguistics	Build a corpus of novel translation Corpus analysis Visualization and report writing.
**6**	Week 11 and Week 12	Mini Project I: Subtitle Corpus	Scrap bilingual subtitles of top 250 movies from online databases, and build a corpus of subtitles from the obtained data
**7**	Week 13 and Week 14	Mini Project II: CAT	Learning CAT fundamentals Feed CAT systems with scraped language data Building a Memory Bank for a specific topic Using CAT for translation in real world.
**8**	Week 15 and Week 16	Mini Project III: A DIY project	Use acquired knowledge for a tiny DIY project. Collaborations beyond groups are welcome. The project should not be similar to any previous tasks. Creativity is welcome and will be rewarded.

Task 1 is the course opener and a primer in data science. Getting familiar with HTML is a good start point for learners with no prior experience in technology because it is straightforward and relatively simple to learn (Fajfar, 2016). Students will be instructed to know the difference between text editing and “WYSIWYG” (what you saw is what you get, like Microsoft Word) editing, and the basic knowledge of structured text in HTML editing. The tasks for students include parsing and editing html files, creating webpages of their own biography and converting texts into CSV files. The task is inspired by the guides of webpage making and editing from *Head First HTML and CSS* (Robson and Freeman, 2012). The aim of the first task is to provide students a gateway toward better understanding of the tasks they are expected to handle in future and necessary basic knowledge to get their appetite whetted.

Task 2 and task 3 are about Python programming. Python has become a dominant force in data science, and is expanding its territory toward fields like social science or digital humanities (McLevey, 2021). Teaching basic programming to students with no prior experience within 2 weeks is challenging. Hence, the teaching materials are limited to a narrow but well-selected scope. In classroom instruction and demonstration sessions, the lecturer starts by showing students how to use Python for simple tasks such as doing math, automating spreadsheet editing, etc. The most important topics to cover in teaching Python for translation trainees are natural language processing with NLTK or similar packages and analysis of text data. Contents for task 3 are relatively more challenging for novice learners, with web scraping and data visualization as the core learning objectives. After the classroom instruction and out-of-class drills, students are expected to know how to use Python to collect and process text data from the Internet.

Task 4 is a direct continuation of previous tasks, and it asks students to use existing API (Application Programming Interface) provided by major online machine translation service providers such as Google Translate or Microsoft Bing for automatic translation. Students are expected to learn the basics of how to make HTTP request and how to parse HTTP response. Students are further encouraged to write their own API codes with production-ready web frameworks such as Flask or Django. Alongside with the instruction and demonstration of API construction and application, students are provided with knowledge on machine translation. Students are requested to inquire into the history of statistical machine translation and understand their impact on the language service industry from a professional angle.

In task 5, students will learn the basics of corpus linguistics. After a brief introduction to the history of corpus linguistics and its significance to the digital world, students will be instructed by the lecturer to build and use corpus from scratch. Prior knowledge of web scraping and natural language processing will be pivotal in this task. Students are encouraged to choose the specific field or topic that interest them most for the data collection and corpus construction. In this task, students are requested to use and report the corpus they have built. Students are encouraged to study the structured text format used for CAT systems. Later, they will be instructed to convert from a certain format (such as excel spreadsheet or SQL database) into the desired format for CAT systems (such as TMX format used by Trados).

Task 6 to task 8 are independent projects for students to use acquired knowledge in simulated situations. The tasks involve building a relatively larger corpus than that of task 5, learning to use CAT systems with collected data, and creating an amateur learning artifact as a summary of the learning outcomes. In the last three projects, students are demanded to wield their creativity and learning ability to finish these tasks. Students are invited to collaborate and practice.

The official version of the course would be available for translation trainees since September 2022. The course would be taught by three lectures with the help of a course coordinator. The information of the faculty members for the course is shown in Table 2.

**Table 2:** Information of course instructors and coordinator.

Pseudonyms	Gender	Age	Educational background	Expertise	Responsibilities in the course
Ins. A	M	35	Master in Translation	NLP, Data Science	Main Instructor.
Ins. B	F	30	Master in Translation	CAT, Data Visualization	Instructor mini project I&II.
Ins. C	F	32	Master in Linguistics	Corpus Linguistics	Instructor of Corpus Linguistics.
Coord. A	F	44	Master in English Language Studies	Translation Education, Educational Psychology	Maintain quality and consistency.

## Materials and methods

### Design

The study adopted mixed method approach by employing survey and focus group discussions as research methods. The rationale for adopting mixed-method research design was to compensate the weakness of qualitative or quantitative research method with the advantage of its alternative (Creswell and Plano Clark, 2018).

During the pilot evaluation, an eight-week special edition of the data science course was used. The reason for adopting a condensed version of the course was the time constraints. The condensed version retained the essential components of the original course. Some of the classroom instruction and demonstration parts were provided as reading materials to students for self-learning. Table 3 shows details of the condensed version of the course for pilot evaluation.

**Table 3:** Course specification (pilot evaluation condensed version).

No.	Week	Task	Contents
**1**	Week 1 and Week 2	Python (I)	Learning Python basics Code to generate html Use NLTK package to analyze text Using Pandas to analyze numeric data.
**2**	Week 2 and Week 3	Python (II)	Web scraping with requests and lxml Scraping COVID-19 data from news websites into spreadsheets or csv files Analyze COVID-19 data Data visualization of COVID-19 data.
**3**	Week 4 and Week 5	Corpus Linguistics	Build a corpus of novel translation Corpus analysis Visualization and report writing Using Flask to write API service of corpus query.
**4**	Week 6	Mini Project I: Subtitle Corpus	Scrap bilingual subtitles of top 250 movies from online databases, and build a corpus of subtitles from the obtained data
**5**	Week 7	Mini Project II: CAT	Learning CAT fundamentals Feed CAT systems with scraped language data Building a Memory Bank for a specific topic Using CAT for translation in real world.
**6**	Week 8	Mini Project III: A DIY project	Use acquired knowledge for a tiny DIY project. Collaborations beyond groups are welcome. The project should not be similar to any previous tasks. Creativity is welcome and will be rewarded.

### Participants

Participants of the study were trainee students in the BTI program at the university. Senior students were excluded from the population due to their relatively rich experience in translation technology and lower level of participation interest caused by graduation pressure. An email letter was sent to all potential participants of the BTI program (total *n* = 368) describing the research specifications, including the purpose of the study, the duration of the pilot evaluation, expected outcomes, course structures, risk and benefits of participation, and anonymity and confidentiality of data, etc. Eighty-five students (*n* = 85) eventually participated in the study voluntarily. Table 4 shows the demographic information of the participants for the present study.

**Table 4:** Demographic information of participants.

Grade	Male	Female	Total
Freshmen	6	17	23
Sophomores	20	24	44
Juniors	7	11	18
Total	33	52	85

### Instruments

For the quantitative strand of the study, a course satisfaction survey was conducted. The survey was adapted from Course Satisfaction Questionnaire (CSQ; Frey et al., 2003; Oyelere et al., 2021). According to previous studies, the CSQ was reliable and internally consistent with a Cronbach’s alpha of ~0.97 (Wang et al., 2013; Oyelere et al., 2021). In the present study, the Cronbach’s alpha value of the survey was 0.96.

To ensure validity, the translation and adaption process in the development of the survey were monitors and peer-reviewed by a panel of experts with curriculum development experiences and expertise. The survey is composed of 21 items which covered multiple aspects of the implementation of the course including the interaction (items 1–3), contents (items 4–9), tasks (items 10–15), teaching styles (items 16–19) and the learning outcomes (items 20–21). Table 5 lists items and sections of the survey.

**Table 5:** Specification of items and sections of the survey.

No.	Item	Section
1	The amount of interaction between you and your instructor	Interaction in learning
2	The quality of interaction between you and your instructor	
3	The cooperation between you and your classmates	
4	The manner in which the tasks of the course were distributed	Contents of the course
5	The logical organization of the course content	
6	The flexibility given to you to complete the tasks	
7	The manner in which guidelines were given on the completion of tasks	
8	The lecture notes and learning materials provided to you	
9	The extra learning resources provided to you (source code and dataset for experiments)	
10	The format of the different tasks	Tasks of the course
11	The learning value of the tasks	
12	The options available to you to hand in tasks	
13	The time it took for your instructor to provide feedback on graded tasks	
14	The quality of the feedback provided on graded tasks	
15	Access to your performance rating during the course	
16	The teaching style of your instructor	Teaching style
17	The assistance given by the instructor in completing the course successfully	
18	The instructor in terms of his devotion to the course	
19	The accommodation of your approach to learning in the way this course was taught	
20	The increase in your digital competence in translation learning as a result of this course	Learning outcomes
21	The increase in your confidence in using the knowledge to solve translation problems as a result of this course	

For the qualitative strand, focus group discussion was used to collect students’ attitudes and perceptions about the pilot course. A focus group protocol was developed by the researchers and peer-reviewed by faculty members and experts. The protocol outlined the recommended procedures for focus group and included pre-determined questions to facilitate and guide the discussion. Table 6 shows questions related to the “suggestion for the course” part of the focus group discussion.

**Table 6:** Sample questions from the focus group protocol.

Item no.	Question
3.1	What part of the course should be improved urgently? The assessment part of the course should be improved.
3.2	In addition to the improvement in course assessment, what corresponding changes should be made in the curriculum to support the change?
3.3	Any suggestions on minor changes to the course? Minor changes mean no dramatic change to the course structures.
3.4	If you were the course instructors, what changes you would bring into the teaching and learning in the official version?

### Procedures

To collect quantitative data for the study, all participating students were invited to finish the satisfaction survey of the course. The survey was administered face-to-face in small groups (6–8 respondents for each group) to minimize the exchange of ideas among respondents. Responses were scored on a seven-point Likert scale, from 1 (totally dissatisfied) to 7 (totally satisfied). Higher score in the response indicated higher level of satisfaction toward the design and implementation of the course for evaluation. After data cleaning, two students’ responses were excluded for giving full scores (7) to all the items.

The collected and cleaned survey data were analyzed with R statistics software. Tidyverse (Wickham et al., 2019) package was used for data analysis. Descriptive statistics were used to investigate the satisfaction level of participants.

The focus group were conducted after the pilot evaluation. To ensure the trustworthiness of the focus group discussion, in-depth training of moderators and the monitor of experts were used. Two lecturers, well informed of the details of the study and well-trained by the researchers, were recruited to serve as moderators for the focus groups. A five-member expert panel consisting of decision-makers of the BTI program and deans of *the School* were invited to monitor and review the procedures and the data produced in the focus group discussion. The focus groups lasted for 45 to 60 min per session, with all discussion and procedures audio recorded and transcribed verbatim. Before the completion of each session of focus group discussion, the field notes and brief summary of the contents of the discussion were reviewed by representatives of the respondents.

Transcribed and cleaned data from the focus groups were analyzed thematically in accordance with the methods proposed by Braun and Clarke (2012). First, the researchers gathered and get familiar with the transcription and field notes. Expert panel was invited to participate in the initial discussion about the comprehensiveness of the data. Second, initial codes were generated from processed data. Two lecturers (Lecturer A and Lecturer B) were recruited to assist the initial coding with the researchers. Third, the researcher searched for themes among generated initial codes. The expert panel monitored the screening and selection of themes. Fourth, synthesized themes were reviewed by the researcher and the expert panel. Fifth, themes were refined to identify the essence and subthemes were synthesized and reordered. Finally, the findings from the focus group discussion were reported for the following procedures of the present study.

## Results

### Results of satisfaction survey

The overall average score of satisfaction for the course was 5.39, indicating a satisfaction level between “slightly satisfied” and “very satisfied” out of a seven-point Likert scale. Average scores for five sections of the survey were 5.44 for interaction (items 1–3), 5.42 for contents (items 4–9), 5.18 for tasks (items 10–15), 5.47 for teaching styles (items 16–19) and 5.69 for the learning outcomes (items 20–21) respectively. Table 7 shows the descriptive statistics of the survey results.

**Table 7:** Descriptive statistics of survey results.

Survey item	Avg	SD	Min	Max	Median
*Section: Interaction*	*5.44*	*1.05*			
Q1: The amount of interaction between you and your instructor	5.54	1.10	4	7	4
Q2: The quality of interaction between you and your instructor	5.98	0.74	5	7	6
Q3: The cooperation between you and your classmates	4.81	1.32	3	7	5
*Section: Contents*	*5.42*	*1.13*			
Q4: The manner in which the tasks of the course were distributed	5.76	1.04	3	7	6
Q5: The logical organization of the course content	5.75	1.13	3	7	6
Q6: The flexibility given to you to complete the tasks	4.60	1.20	3	7	4
Q7: The manner in which guidelines were given on the completion of tasks	5.42	1.22	3	7	6
Q8: The lecture notes and learning materials provided to you	5.47	1.03	3	7	6
Q9: The extra learning resources provided to you (source code and dataset for experiments)	5.53	1.16	3	7	6
*Section: tasks*	*5.18*	*1.15*			
Q10: The format of the different tasks	5.44	1.19	3	7	5
Q11: The learning value of the tasks	5.92	0.95	3	7	6
Q12: The options available to you to hand in tasks	4.54	1.26	3	7	5
Q13: The time it took for your instructor to provide feedback on graded tasks	4.82	1.19	3	7	5
Q14: The quality of the feedback provided on graded tasks	5.27	1.20	3	7	5
Q15: Access to your performance rating during the course	5.08	1.14	3	7	5
*Section: Teaching Style*	*5.47*	*1.21*			
Q16: The teaching style of your instructor	5.74	1.12	3	7	6
Q17: The assistance given by the instructor in completing the course successfully	5.71	1.15	3	7	6
Q18: The instructor in terms of his devotion to the course	5.65	1.28	3	7	6
Q19: The accommodation of your approach to learning in the way this course was taught	4.80	1.30	3	7	5
*Section: Learning Outcomes*	5.69	1.12			
Q20: The increase in your digital competence in translation learning as a result of this course	5.74	1.10	3	7	6
Q21: The increase in your confidence in using the knowledge to solve translation problems as a result of this course	5.65	1.13	3	7	6
Average	5.39	1.13			

For satisfaction in the interaction between students and instructors during the pilot course, students favored the quality (avg = 5.98) and amount of interaction with instructors (avg = 5.54). Given the limited number of instructors involved and the challenges of the contents of the course, the instructional quality was a strength of the course at current stage. However, students’ satisfaction in in-class interaction with peer students and out-of-class interaction with group members were relatively lower (avg = 4.81).

For the satisfaction of contents of the course, students reached a consensus, except for the flexibility given to them in finishing the tasks (avg = 4.60). Students were affirmative in the way the course was designed (avg = 5.76) and the way contents were distributed throughout the syllabus (avg = 5.75). Richness in materials provided in-class (avg = 5.47) and out-of-class (avg = 5.53) and support from instructors to finish the tasks (avg = 5.42) were well-acclaimed. The issue of relatively lack of flexibility, based on students’ review, should be studied in following parts of the study.

Students were positive in the value of the tasks within the pilot course, but they were not satisfied with the efficiency of feedback and grading from instructors (avg = 4.82) and the availability of learning artifact submission channels (avg = 4.54). The relatively slow in response to students’ performance could be a result of shortage of hand put into the pilot course. From a faculty mostly composed of lecturers with translation and English language educational ground, recruitment of competent instructors was challenging. The issue could be solved by providing technology related training for in-service lecturers.

Students were mostly satisfied with the teaching style of the instructors (avg = 5.74), but their adaptation to the task-based learning approaches implemented in the pilot course was far from satisfactory (avg = 4.80). During the pilot course, basic data science and programming skills were taught in an interactive manner with live coding and demonstration of application of modern technology in translation. The teaching was effective and appealing to most students. However, the seemingly incompatibility of students’ own learning style within the pilot course needed further inquiry.

In regard to learning outcomes, feedback from students were promising and encouraging. It could be concluded that most students were satisfactory with the data science knowledge taught during the pilot course and were contented with the enhancement in digital competence (avg = 5.74) and the mastery of translation technology (avg = 5.65) after the pilot course.

### Students’ attitudes and perceptions toward the course

Thirty-five students voluntarily participated in the focus group discussion, hence, six focus groups were undertaken (n = 5, 6, 5, 7, 6, 6). Majority of the students taking part in the focus group discussion were female (*n* = 31, 88.6%). More sophomore and junior students (*n* = 27, 77.1%) participated in the focus group. Students were aged between 18 and 22 (average = 20.2 years).

The analysis of the focus group identified four main themes related to the experience from the pilot evaluation: (1) gains from the course (2) challenges of the course; (3) task-based learning approach; (4) suggestions for improvement.

#### Gains from the course

When asked about the gains from participating the pilot project, students voiced their achievement in both knowledge and understanding from various perspectives. Three subthemes were synthesized from the transcribed and coded data.

##### Digital literacy

To participants of the pilot project, the gains in digital literacy was generally agreed upon. Students reported that their level of digital literacy was promoted after the pilot project. Their abilities to use digital resources and digital tools to solve encountered problems were stronger than before. The enhancement in digital literacy was not limited to the field of translation technology. As reflected by a male student, the gains in digital literacy were “general for many aspects of learning and life.” He said that his knowledge in using common internet tools grew after the pilot project, for example, he can “use advanced keyword and search strategies” to accurately search for needed information. A significant boost in learning and working efficiency was confirmed by participants, especially in dealing with tasks calling for higher command of digital capabilities.

##### Useful techniques

Many tools and techniques were praised by participants for their applicability and strength in solving problems related to translation. Among them, the following were highlighted by participants of the pilot evaluation: corpus, text mining and analysis, and visualization.

First, students reported that the construction and application of corpus was frequently practiced on their own after the pilot evaluation. Based on their views, corpus was a versatile tool in many fields such as text analysis, lexical analysis, and stylistics studies. The construction of corpus needed basic data retrieval, data cleaning and data processing abilities, which were the core competence prescribed in the pilot course.

“… corpus… is really useful. I use it a lot in other courses as well. For example, in the literature translation class, I used it to study styles of different translators. The lecturer gave me very high marks…”

Second, text mining and analysis was mentioned by respondents frequently during the focus group discussion as a primary gain in knowledge during their participation in the pilot project. The claim from a male student that “students should be able to get text and materials from the internet instead of just textbooks” received approbation from peers during the focus group session. Based on the synthesized results from the transcribed discussion, the primary usage of text mining technique for translation learners at undergraduate level includes (a) retrieve text data from more sources; (b) extract characteristics of text data and report statistically; (c) find latent ideas and attitudes behind text. As reviewed by a participant after the pilot study:

“… text mining is cool. In our tasks, we use the scraping and mining tools to study the geographical distribution of negative comments for a product during the pandemic… It’s a joy to use such tool …”

Finally, data visualization was favored by many participants. According to the respondents in the focus group discussion, the strength of data visualization was new and effective for translation students. Its ability to present characteristics of text in an appealing manner is attractive. Several participants reported their own application of data visualization in other courses:

“… I like the lecture note from first glimpse for the colorful and informative charts… I used it with text analysis in my semester paper for the course of ‘audiovisual translation’. In the paper, I studied lexical features of two movies…”

Other gains such as automation of tedious and repetitive tasks, reporting findings in multimodal formats, and building web applications were mentioned during the focus group discussion. However, these techniques were less frequently mentioned and received little consensus from peer respondents.

##### Interest in the field

When asked about the interest in data science after the pilot evaluation, participants generally showed positive feedback. Their interests were reflected in their ambition to continue learning related courses, outlook for a future career relevant to language data science, interest to use acquired knowledge for graduation thesis, and desire to continue postgraduate study in relevant fields. A female student whose graduation thesis was on subtitle translation and corpus linguistics wrote in her acknowledgement:

“… the pilot course of language data science was an eyeopener for me… My decision to use corpus linguistics to study subtitle translation was made just after the course. I think it’s a perfect combination of two of my interest…”

#### Challenges of the course

When asked about the difficulties of the course, students gave varied opinions. Based on their understanding, the most difficult part of the course is the “shift of mind,” a process to gain confidence and build competence in programming and data analysis. Following four subthemes were synthesized from the transcribed focus group discussion.

##### Prerequisite knowledge

In regard to the difficulties in learning caused by lack of prerequisite knowledge, students voiced different opinions. It should be noted that the participants include students who have finished the CAT courses. For those students, the data science course was easier to get started with, and the learning seemed more like a continuation of “where we stopped in the CAT course.” For freshmen who had little experience with translation technology, their experiences were different. As a female students concluded in the focus group:

“… I always regard myself as a student of arts instead of science, and my experiences with computers were rather limited… I think the difficulties in the course was caused by shortage of a supportive course that can let us know what we are doing in the first place…”

##### Computational thinking

A consensus was agreed upon by participants that the shift toward “computational thinking” was the most challenging. According to the respondents, the lack of “DIY spirit” and the abilities to solve problems by using the computer properly contributed to their feeling of “being shocked yet amazed” after the initial sessions of the course. The shift in mind toward a “coding thinking” or “computational thinking” took time. However, many students agreed that they eventually “got it.” Some participants also mentioned a sense of less “preparedness.” As reflected by a male student:

“… the moment I want to start coding (even though very simple) was like the moment when I sat in front of a piece paper and was asked to write down a Chinese classic essay by memory…”

##### Lack of confidence

Some participants reported a lack of confidence in completing some of the most challenging tasks in the course. Some of the tasks were complex and difficult for undergraduate students and student had to spend rather longer period of time than expected to solve the problem. According to many respondents, repetitive attempts to debug errors in their snippets of code often resulted in lower self-confidence. As reflected by a participant:

“I have to admit that error symbols and messages from text editors were really hurtful, especially when you felt you were confident enough…”

The lack of confidence was also caused by the shortage of a platform for them to discuss and exchange ideas in learning. Students claimed that in a task-driven learning environment, lecturers should also take care of their emotional and motivational status. The relative longer period of time spent to finish the task caused “stress” and “academic burnout” among participants.

##### Incremental difficulties

When asked about difficulties between different sections of the course, many students confirmed that the incremental design in course content ensured a generally satisfactory learning experience during the pilot course. Based on the observations of students’ performance and the feedback received during the course, many students agreed with the claim that “the initial phase of the course was challenging, and the midsections were generally smooth with great inner-consistency, while the finishing parts were again hard to grasp and very challenging.”

Nevertheless, the proportion of tasks needs coding raised controversy among participants. Some students were satisfied with the frequency of coding during the course, while others were doubtful. The idea of course developers to uphold the “starting from scratch” spirit in technology learning was challenged by participants for being “unrealistic” and “too idealistic.” A female student wrote the following reflection in her course summary notes:

“I like the idea of teaching us coding, but I only wish it could be controlled to a more measured amount. The teacher himself also said that we have alternatives and mature tools for many of the tasks. So why not use them?”

#### Task-based learning approach

During the focus group discussion sessions, the researchers and the moderators followed closely to the focus group protocol to keep the discussion focused. The questions on the protocol were to understand students’ attitude and perceptions on the gains, challenges, significant and suggestions for the course. However, students discussed occasionally about their learning experiences in the task-based learning environment during the course. The following subthemes were synthesized from the discussion transcription:

##### Engagement

When asked about the learning experiences, students claimed that the task-based model was effective in raising their interest and keeping them engaged in learning. Students generally showed satisfaction about the task-based learning implemented in the course, as a female students reviewed:

*“… I like that the course is made up of several individual tasks. Each task has its own focus, but they are internally connected as well … The tasks made us interested and focused on how to solve the problems with knowledge learnt in class and out of class as well …”*

Based on classroom observations, students were more involved in classroom activities during the course. Volunteering participation in classroom interactions and presentation of learning artifacts were popular. The data science course has formed a “it’s natural to err for beginners” atmosphere which is friendly to students with less confidence. As a male student remarked on social media that “even the lecturers made mistakes with live coding sessions, why should I fear making mistakes”? After viewing the classroom teaching from video recordings, the BTI program leader voiced her reflections about in-class learning engagement:

“It’s pretty encouraging to see the changes the task-driven learning environment has brought. Students were proactive to show their own progress and insights.”

For out-of-class learning activities and knowledge sharing, students have reported high level of engagement and interest. Student believed that the tasks-driven learning model effectively kept them interested and focused. Based on the observation of out-of-class discussion in person and in online learning groups, students were willing to compete with other groups in solving problems. On the night of 15 December 2021, a students commented on the ongoing discussion in learning groups on instant messengers:

“… so strange you guys are still learning at this time (10:20 pm approximately), and here is our solution…”

##### Teamwork

Students agreed that team collaboration was pivotal for the course. In some groups, all tasks were completed with genuine teamwork, in which members equally shouldered the responsibilities for a part of the task. However, there were some criticisms about the teamwork collaboration. A well-supported complaint was about the unequal digital abilities among group members. This was believed to be a major reason for differences in task-completing efficiency. The issue partly contributed to the relatively lower level of satisfaction in “Q3: The cooperation between you and your classmates” in the survey.

A very unexpected finding was that students autonomously formed informal learning groups during the pilot project. The starting point is that students were willing to continue learning by going beyond the pre-determined groups for classroom activities. Some students with relatively higher level of digital literacy and computer skills were popular. Later on, several informal learning groups and online learning communities were formed, in which students arrange and regulate learning on their own. The informal learning experience remain unknown to lectures until the focus group discussion sessions.

##### Task-based learning

When asked about the learning experiences during the pilot course, students indirectly showed their understanding of task-based learning. First, students understood and supported the multistage design for each task. In the learning artifacts and task completion presentations, many groups divided their reflection of learning experiences and procedures into pre-task, task and post-task phases. The division was in line with the basic outline of a task-based learning task. Second, students emphasized the importance of preparation and reporting stages of each task. Students agreed that in the preparing and summarizing stages, they were provided opportunities and resources to learning by themselves and report the learning artifacts and results in the most creative way. Finally, students were positive toward the modular learning design of the course. As a student remarked in his semester report that “the course generally feels like a game in which you upgrade and earn experience step by step.”

#### Suggestions for improvement

When offered the chance to suggest for improvements, students gave a volume of useful suggestions. Five subthemes were extracted from synthesized transcription of focus group discussion and project reports.

##### Extend course duration

During the focus group discussion, many students suggested that the length of the course should be extended. However, this was due to time constraints to evaluate the course. Students no longer worried about the length of the course after being told of the length of the official version of the course.

Without changing the duration of the course, students suggested alternative teaching and learning approaches could be included in the official version of the course. When asked about the possibility to use informal learning and community learning to supplement the teaching and learning of data science, students favored the suggestion in general. Some students suggested that out-of-class learning should be encouraged and supported in all courses in future.

##### Reorder the technology courses

Bearing in mind that the BTI curriculum included several other technology courses, students suggested that the ordering of technology courses could be reconsidered. According to the original ideas of course developers, the course of data science would be the first one out of a technology curricular track. The student believed that if the data science course followed a traditional translation technology course, for example CAT or MT, it would be easier for students to comprehend. Additionally, if reordered, the complaints about the difficulties of the course would be remedied as students were more experienced with translation technology and state-of-the-art tools for data processing.

##### Lower the difficulties

Apart from above-mentioned suggestion to reorder the sequence of technology courses, students made substantial suggestion to lower the difficulties of the course.

In regard to the contents of the course, students agreed that the Python programming part of the course was rather challenging, especially when students were of little previous experiences in related fields. Student expressed expectation in learning basic programming knowledge for advanced data science techniques. However, suggestion was made to replace part of the programming teaching with existing software or low-code platforms.

##### Enrich learning resources

Students advised the course developers to enrich pedagogical resources and improve learning environment for the data science course. Suggestions were also made in upgrading learning platforms and communication platforms.

First, the learning resources available for the pilot course was rather limited. A contradiction was observed by comparing the results of the survey and analysis of students’ opinions (as in *Q9: The extra learning resources provided to you* from the survey). It indicated that students showed understanding for the issue since the course was developed and evaluated recently. However, more supplementary learning resources and alternative solutions to problems should be included in future.

Second, similar voiced were heard in regard to the frugal set up of learning environment. Confided by out-of-date devices and pedagogical instruments available for the BTI program, students were unsatisfactory about their learning experience. Student suggested that more learning approaches and teaching models could be considered to remedy the rather “disappointing” learning environment.

Finally, better learning and communication platforms could be provided to students. The claim supported the observed lower level of satisfaction in options in handing learning artifacts (as *Q12: The options available to you to hand in tasks* from the survey). Students suggested several alternative code sharing and knowledge sharing platforms for consideration in an instant messenger conversation:

“Student A: I suggest using Github for code sharing. All of us can fork and reproduce the code from others.
Student B: I strongly disagree, Github may bring extra burdens to those who have no idea what it is about.
Student A: But the convenience it brings is huge.
Student C: … Dingding is good enough.”

##### Integrate better in the curriculum

Better integration with other courses and training workshops were expected from students. Reviewing the existing curriculum, the teaching and learning of translation technology were scattered in different courses. A refreshed curriculum with integrity was desired by students to lower the difficulties in acquiring needed knowledge in a more consistent manner. The idea was reflected in the remark of a male junior students who has experienced both the CAT course and the data science course:

“… in the CAT course, the lecturer was very ambitious telling us that a new course that will teach us how to scrape and process data would be available in the coming semester… now we have it… I truly hope the two courses could be inter-connected better in the future… and that will increase its strength enormously…”

To ensure better integration, the competence of teachers was also a concern. Students suggested that teachers could be trained in advance so that the teaching and learning of different technology courses would be in line with each other. For example, in the CAT course, the lecturer used Trados as the main software while in the data science course, the lecturer adopted MemoQ instead. Additionally, the attitude of using state-of-the-art tools and technologies in the data science course was not shared by other lecturers. According to a female participant, “… some of our courses use very old-fashioned technologies … it would be better if they get updated … This would help us greatly when we enter the job market.”

## Discussion

The pilot evaluation of the course of data science for translator demonstrated that the efforts to cultivate students’ digital literacy and technology competence through teaching data science was well-received by the participants. In the present study, students received an 8-week condensed session of a data science course for translators. The course covered introductory knowledge of data science, natural language processing, and the application in text analysis for translators. Participants generally were satisfactory with the contents and the design of the course. Students reported high level of involvement in learning data science techniques and strong interest in applying acquired knowledge to solve simulated problems. Participants expressed their willingness to continue the learning of data science and translation technology as academic or career pursuit for the future. In addition to the evaluation of the pilot course, the findings of the study indicated positive effects of task-based learning approach in enhancing students’ learning engagement and interest. With the initial success in evaluating the course, the findings and experiences of from the development and evaluation of the course brought new insights into the teaching and administration of translation education.

### Implication on translation education

The development and evaluation of the pilot course of data science for translators was a primary effort to innovate on existing translation technology courses in *the university*. With its phasic success, the team would look forward to further changes in teaching translation at undergraduate level.

First, the contradiction between translation education standards and the demand for high-quality translators with good command of technologies should be addressed. In the pilot evaluation, respondents expressed their understanding, expectation and preference in learning modern translation technologies. The opinions and attitudes from participants were in tandem with the current trend in the development of translation education. The constituents of translation competence have been dynamic in answer to social and technological changes of the current era (Pym, 2011; Marczak, 2018; Wang, 2019). According to Pym (2014, 489) “information mining competence” and “technological competence” should be included as components of the overall competence required for translators. The study demonstrated that the inclusion of translation technology as a subcategory of translation competence is well-supported by students who had finished the course.

Nevertheless, In the recent development of China’s English ability rating scale (in which translation competence is a subcategory), translation technology competence and digital literacy of translators or translation trainees are still not clearly defined (Jin et al., 2017). In many undergraduate translation training programs among Chinese universities, the position of technology competence or digital literacy were greatly underestimated. As a human resource manager for a language service company said during an interview:

*“We truly welcome students with good computer skills … but every year we have to retrain them … we think universities should pay more attention to this”* (J. Li. 2021, interview with author, September 15).

Second, innovation in translation technology course development should be encouraged. In the pilot evaluation, students showed their interest in learning the newest technologies and generally gave good performance in completing the tasks. The outcomes of the course were contrary to the doubts and mistrust faced when the course was developed. The initial success in the pilot evaluation suggested the possibility to redesign translation technology courses with boldness and creativity. Take *the university* for example, existing CAT course, according to the original course developer in a message to the authors, was to “let students’ know basic knowledge of CAT” (Y Gao 2021, personal communication, 12 June). The underestimation of students’ potential to make progress in grasping technology largely limited the value and impact of the course in shaping the students’ comprehensive abilities as future translators. The present study revealed that students were capable to learn and master technology, even the latest ones. Top students performed outstandingly with creative and fancy learning artifacts at the end of the pilot course (see Supplementary Appendix 2 for the screenshot of a small piece of software for auto translation with graphical user interface). However, throughout universities providing accredited translation education in China, less than 5% of them are providing courses and instructions beyond the scope of CAT (Wang et al., 2018). It is urgent to broaden the scope of translation technology courses, as “apart from CAT” translators “often have other tasks to be performed with various software” (Dabis, 2020, 104). The design of translation technology course should be in line with the trend of cutting-edge technologies used in language service industries and the demands from job market. At present, it is a good opportunity to redesign translation technology courses and curriculum.

### Recommendations for decision-makers

Upon the reflection of the gains and pitfalls during the development and implementation of the course, the researchers would change the perceptions of translation education in the eyes of educational administrators and university decision-makers. Based on the suggestions from students during the pilot evaluation, following recommendations were made: (1). encouraging alternative learning approaches; (2). improving teaching and learning environment for BTI programs; (3). retraining translation trainers.

First, decision-makers and BTI program directors should encourage the adoption of alternative learning approaches. Throughout China, translation education uses lecture-centered teaching model. However, the learning outcomes and engagement of trainee students were not guaranteed, comparing to those from an alternative learning environment. For courses like translation technology or computer-aided translation, the confinement in learning approaches would result in extensive proportion of theoretical teaching, which means a direct curtailment of time and chances for students to practice. In the study, by implementing task-based learning, students were offered opportunity to use their creativity and learning ability in exploring the world of data science. Consequently, the learning process proved efficacious in enhancing their agency and motivation in knowledge acquisition. The researchers of the present study would call for the embrace of alternative learning approaches in translation education.

Second, learning and teaching environment for BTI program should be updated. The shortage of pedagogical instruments and devices related to translation technology should be addressed immediately. In the pilot evaluation, a significant part of suggestions for improvement were related to the shortage of tailored learning environment. The issue was partly caused by the stereotypical perception that translation education was merely a “special form” of language education. As researchers seeking innovation in education, we encountered misunderstanding and obstacles from beginning to the end from university administrators. For example, in searching for appropriate site for the pilot evaluation, the administrators were reluctant to allocate computer laboratories for the project, claiming that translation learning was not “in need of laboratories.” Lecturers and learners in the contemporary era need not only language laboratories and multimedia classrooms, but also state-of-the-art pedagogical instruments and learning environments. The call for upgrades in devices and augmentation in learning environments should be supported to elevate the quality of translation education.

Finally, the shortage in talents capable of teaching and instructing students’ learning of modern translation technology was a common problem shared by many institutions (Cui, 2017). Only 6.25% percent of lecturers from Chinese translation training programs were from a computer science or interdisciplinary educational background (Wang et al., 2018). In the pilot evaluation, students reported lack of consistency between different translation technology courses in regard to adoption of technology and tools. Efforts should be made to train in-service and pre-service translation trainers for up-to-date digital competences.

### Limitations and future perspectives

The limitation of the study included relatively limited number of participants in the evaluation and the limited duration for the pilot course implementation.

Fewer participants recruited from the population threatened the trustworthiness of the findings. Students might opt to not participate in the pilot project due to pressure of routine study during normal academic session. To guarantee the credibility of the study, following measures were taken. On the one hand, the expert panel members who have been actively involved in the development of the data science course continued to help monitoring the evaluation of the pilot course. On the other hand, the course was reduced from 16 weeks into 8 weeks during the pilot evaluation. Confined by a shorter duration of course implementation, the researchers intentionally omitted some of the contents and lowered the complexity of certain tasks. Hence, the quality of the course was under rigorous scrutiny of the expert panel throughout the evaluation.

The study left much space for future research in course development. In the Chinese context, the development of translation technology course was far from satisfactory. Additionally, the connection between technology courses and other courses in the curriculum was weak. Based on the investigation of syllabus and curriculum documents in BTI programs across undergraduate universities in China, only a few institutions clearly listed translation technology competence as a subcategory of the objectives of the program. The findings of the present research proved the feasibility to redesign technology courses in translation education. The course was only one of the redesigned courses for the BTI program. A curricular track for cultivating students’ technology competence was proposed, including the data science course. The motivation for the further development of translation technology courses was to address the absence of systematic curricular tracks of translation technology in Chinese BTI programs. In future, a comprehensive curricular combination would be available for translation trainees at the university. The proposal of the curricular track would be the first step for the BTI program at the institution to shift toward a restructured and competence-oriented curriculum for cultivating job-ready future translators (see Table 8 for the curricular track and related information).

**Table 8:** Proposed curricular track of translation technology.

Courses	Type and duration	Position	Contents	Status
Data Science for Translators	Course(16 weeks)	Introductory	Teaching basic knowledge on data science and digital skills	Finished pilot evaluation
Digital Skills Workshop	Workshop(30 hours)	Introductory	A hand-on workshop to instruct students practicing digital skills	Finished development
CAT and MT	Course(16 weeks)	Intermediate	Introduction to CAT and MT and their application in the language service industry	Finished redevelopment of existing course
Computer-aided Translation and Interpreting Workshop	Workshop(30 hours)	Intermediate	An accompanying workshop for students to practice their CAT and MT skills	In development
Career-oriented Translation Technology	Course(8 weeks)	Intermediate/Advanced	Intermediate/advanced course about new and real-world technologies for job market	Pending approval for development
Pre-service Translator Skill Workshop (including technology components)	Workshop(10 hours)	Advanced	A training session for pre-service translators. Technology related skills are included	Pending approval for development

## Conclusion

The research was the pilot evaluation of a course designed to teach introductory knowledge of data science to undergraduate translation trainees. The participating students gave positive feedback and general satisfaction to the quality and design of the course. Respondents of the focus group expressed their attitudes toward the gains, difficulties and learning approach of the course. Suggestions for improvement were also made by students for the official version of the course. The findings of the study supported the feasibility and significance of providing translation learners with a course of data science for the comprehensive development of digital competence. Additionally, the effectiveness of task-based learning approaches was observed in the pilot evaluation for enhancing students’ learning engagement. The success of the course was an innovative effort to bring about changes to undergraduate translation education in the context of constructing “New Liberal Arts” in China. Future studies would investigate into possible ways to build a comprehensive and consistent curricular track of modern technologies for translation trainees.

## Data availability statement

The raw data supporting the conclusions of this article will be made available by the authors, without undue reservation.

## Ethics statement

Ethical review and approval was not required for the study on human participants in accordance with the local legislation and institutional requirements. The patients/participants provided their written informed consent to participate in this study.

## Author contributions

DY contributed to conception and design of the study, wrote the method section of the manuscript, and performed the statistical analysis. JW wrote results and discussion sections of the manuscript. All authors contributed to the article and approved the submitted version.

## Funding

This research was supported and funded by the Xinyang Agriculture and Forestry University Technology Innovation Team Research Project (XNKJTD-020).

## Conflict of interest

The authors declare that the research was conducted in the absence of any commercial or financial relationships that could be construed as a potential conflict of interest.

## Publisher’s note

All claims expressed in this article are solely those of the authors and do not necessarily represent those of their affiliated organizations, or those of the publisher, the editors and the reviewers. Any product that may be evaluated in this article, or claim that may be made by its manufacturer, is not guaranteed or endorsed by the publisher.
