# Supplementary material for: Teaching data science to undergraduate translation trainees: Pilot evaluation of a task-based course
Source: Front Psychol. 2022 Aug 3;13:939689. doi: 10.3389/fpsyg.2022.939689 (PMC9381704; doi:10.3389/fpsyg.2022.939689)
Supplement: Supplementary file 1 [file Data_Sheet_1.PDF]

Appendix 1– Sample Instructional Material (Building a Corpus of Subtitle Translation)

课程综合练习项目：迷你字幕语料库的搭建和使用

信阳农林学院 闫达

总体思路

本项目组的两名成员，来自信阳农林学院，对于语料库有着很高的热情，但是长久以来，我们的热情都存在于空想阶段和兴趣阶段。机缘巧合之下，我们选择开始我们的实践，我们期望我们一方面可以在研究过程中学到更多，另一方面，也想踏踏实实的建设出来能为更多人使用的特色语料库。如下便是我们在项目初期实现的功能和我们的一些思考的展示。

现有问题

项目组认为语料库研究和发展中存在如下问题：

1. 语料搜集工作过于缓慢
2. 语料整理工作缺乏高度自动化
3. 语料库相关软件平台局限性太强。
4. 语料库软件严重落伍和缺乏更新
5. 语料库与翻译和教学的结合程度不够高。

实现方法构想

项目组拟定使用Clojure程序设计语言，结合JVM平台的优势，实现并发、高速的语料库搜集、整理、查询、存储和与其他领域互通等功能。

最终的成品，应当构架起一套独立的生态系统，源代码开放、跨平台，具有很强的扩展性。

创新之处

本项目组拟定在实现过程中实现如下的创新，并解决1.1中提出的全部预设问题。

1. 将一些具有格式上鲜明特色的文本转化为语料库。
2. 提高专业化文本的语料搜集、整理工作。
3. 构建一套独立的、从零开始创造的语料库生态系统。
4. 为语料库提供更快的搜索和查询方式，例如微信小程序等。

难度所在

从零开始构建生态系统，需要实现和完善语料库和计算语言学框架下许多不同层面的问题。为了提高效率，也许要结合机器学习和人工智能等最新前沿技术，整体开发难度较大。

迷你语料库概览

迷你语料库的目标

本项目为项目组长期项目的起点和一个较小的分支项目，本项目的目标为通过英语电影字幕，整理和生成字幕语料库，以便查询和使用，并对语料库进行语言学和统计学的分析，提高语料库的使用效果。

取得存放的字幕

整个迷你语料库程序以及所使用的字幕，我都已经发在了github上，如下就是这个目前处于**开放状态**的repo。感兴趣的话，可以随时fork和关注，并提出宝贵的意见。😄

因为在repo之中已经生成了部分阶段性的展示结果，因此首先将这些阶段性的成果文件删除，以便下面重新生成。

```
ls /subtitles && rm /subtitles/*.dat && rm /subtitles/subtitles_corpus.png && ec
```

可以看到的是，我们已经将所有的阶段性的成果都删除了。目前只留下了

- clojure语言需要的配置文件 deps.edn（下文可以见到其内容）
- 程序源代码文件夹src
- 字幕存放的文件夹srt
- 语言处理需要的模型目录models

字幕基本知识

这里选择的字幕格式是srt。

> **SubRip** is a free software program for Windows which extracts subtitles and their timings from various video formats to a text file. It is released under the GNU GPL.Its subtitle format’s file extension is `.srt` and is widely supported. Each `.srt` file is a human-readable file format where the subtitles are stored sequentially along with the timing information. Most subtitles distributed on the Internet are in this format.
>
> –Wikipedia

如下是一个韩文的字幕文件：

```
00:03:17,440 --> 00:53:20,375
칠레 한인회에서는  그동안 코로나-19로 인해서 빠트로나또 한인타운가가 5개월 동안 문을 닫은 상[illegible]
정성기 칠레 한인회장님께서 방역 활동을 하고 계십니다.  방역 활동 구역은 만사노, 산타 필로메니[illegible]
황성남 한인회이사님께서도 방역 활동에 동참 해 주셨습니다.


2
00:54:20,476 --> 03:16:22,501
까날 트레쎄 13번 칠레 방송국에서 빠트로나또 개장 현황을 취재하던 중에 한인회의 방역 활동을 목[illegible]
촬영이 끝난 후 인터뷰 요청이 있어서 정성기 한인회장님께서 인터뷰를 하게 되었습니다.
금일 9월7일 월요일 밤 9시 뉴스시간에 촬영 및 인터뷰 내용이 방영 된다고 합니다.
<ref></ref>우리 모두 코로나-19 퇴치 운동에 적극 동참하여 또 다시 자가격리로 되돌아가는 일 [illegible]
```

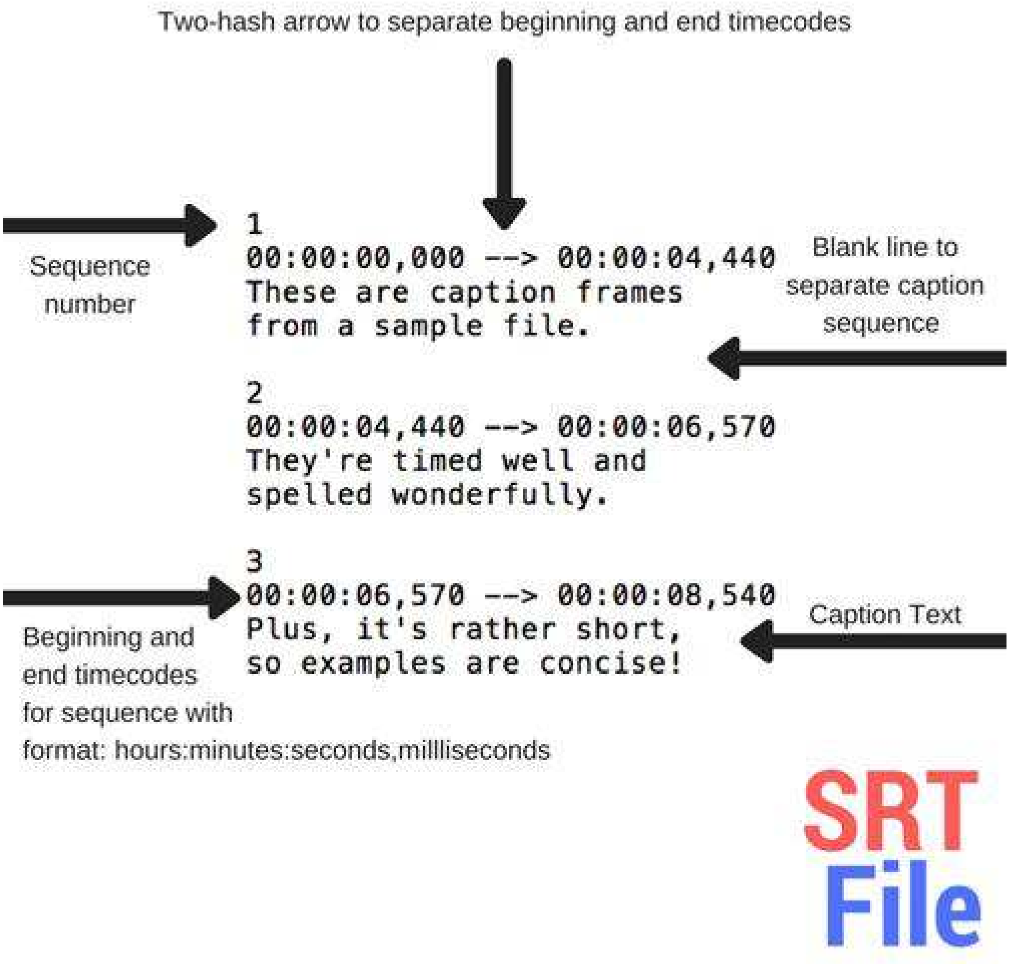

除了文字之外，字幕文件还包括一定的格式。往往借用HTML的tag来实现。

> - Bold – `<b>…</b>` or `{b}…{/b}`
> - Italic – `<i>…</i>` or `{i}…{/i}`
> - Underline – `<u>…</u>` or `{u}…{/u}`
> - Font color – `<font color="color name or #code">…</font>` (as in HTML)

对于语料库的准备而言，这些均属于干扰信息，我们需要将其去除。

我们的语料

是时候看看我们的语料是什么样子了。😸

首先我们来装个软件查看文件，这里使用bat，比系统提供的cat更加强大。😄

```
curl -LO https://github.com/sharkdp/bat/releases/download/v0.16.0/bat_0.16.0_amd
dpkg -i bat_0.16.0_amd64.deb && rm *.deb
```

这里来看看我们的字幕：

为了节约空间，这里只用前300行给大家做一个示范。具体的完整字幕，可以从github的repo获得。不难看出，我们的字幕文件是标准而规范的。

```
bat -r :300 /subtitles/srt/*.srt
```

清理思路

问题认知

文本的清理其实是一个很重要的过程。我们再来看一下我们语料中需要清理的部分。

> 73
>
> 00:05:55,753 --> 00:05:56,903
>
> **Chen Yi's New Fourth Army**

除了黑体部分的内容，其余的都是无用信息。

上面的数字中的第一行，代表电影的场景编号。

第二行中看起来非常复杂的数字是该行字幕出现和结束的时间，字幕文件正式通过这个起点和终点来使字幕有条不紊的出现在电影中。

虽然我们此处搜集的4个电影的字幕文件都没有使用上面说过的格式tag，但是我们要充分考虑兼容性。如果出现格式tag，也许要处理。包含格式tag的字幕行列如下所示：

> 6
>
> 00:02:36,040 --> 00:02:40,158
>
> I'm honoured by my ***comrades'*** nomination...

分析问题

既然需要去除这两点冗余信息，那么我们来分析分析它们吧。

Tag分析

tag的问题非常容易解决，因为tag沿用了HTML的tag格式，所以无论是什么样的格式，tag均由下面的模式构成：

> TEXT

大部分的格式之中，X和后面的/X是一样的，但是其实是否一致根本不重要，对于我们来讲，只需要将这个问题识别为，前后两个由尖括号包围起来的标签，后面一个由/开始即可。

数字分析

通过上文，我们发现，所有的数字都是由场景编号+时间轴起止构成的，两者中间有一个空行。

因此我们可以将这个问题识别为：

> 数字\n两个数字:两个数字：两个数字，三个数字 --> 两个数字：两个数字：两个数字，三个数字\n

这样来看，这两个构成元素完全可以同时处理，因为所有的场景编号和时间轴起止点都是一起出现的，中间有一个空行。

神奇武器登场

问题已经经过了分析，现在是解决的时候了。我们需要使用**正则表达式**来实现我们的目标。

正则表达式简介

正则表达式又称规则表达式。（英语：Regular Expression，在代码中常简写为regex、regexp或RE），正则表达式通常被用来检索、替换那些符合某个模式（或规则）的文本。

正则表达式是对字符串操作的一种逻辑公式，就是用事先定义好的一些特定字符、及这些特定字符的组合，组成一个**规则字符串**，这个规则字符串用来表达对字符串的一种**过滤逻辑。**

正则表达式具有如下的特点：

1. 灵活性、逻辑性和功能性非常强
2. 可以迅速地用极简单的方式达到字符串的复杂控制
3. 对于刚接触的人来说，比较晦涩难懂

我们要做的事情，就是按照正则表达式，将我们预设需要处理的文本进行匹配。从各种各样的情况中，找出一条条能帮我们全面覆盖所有问题的规则。

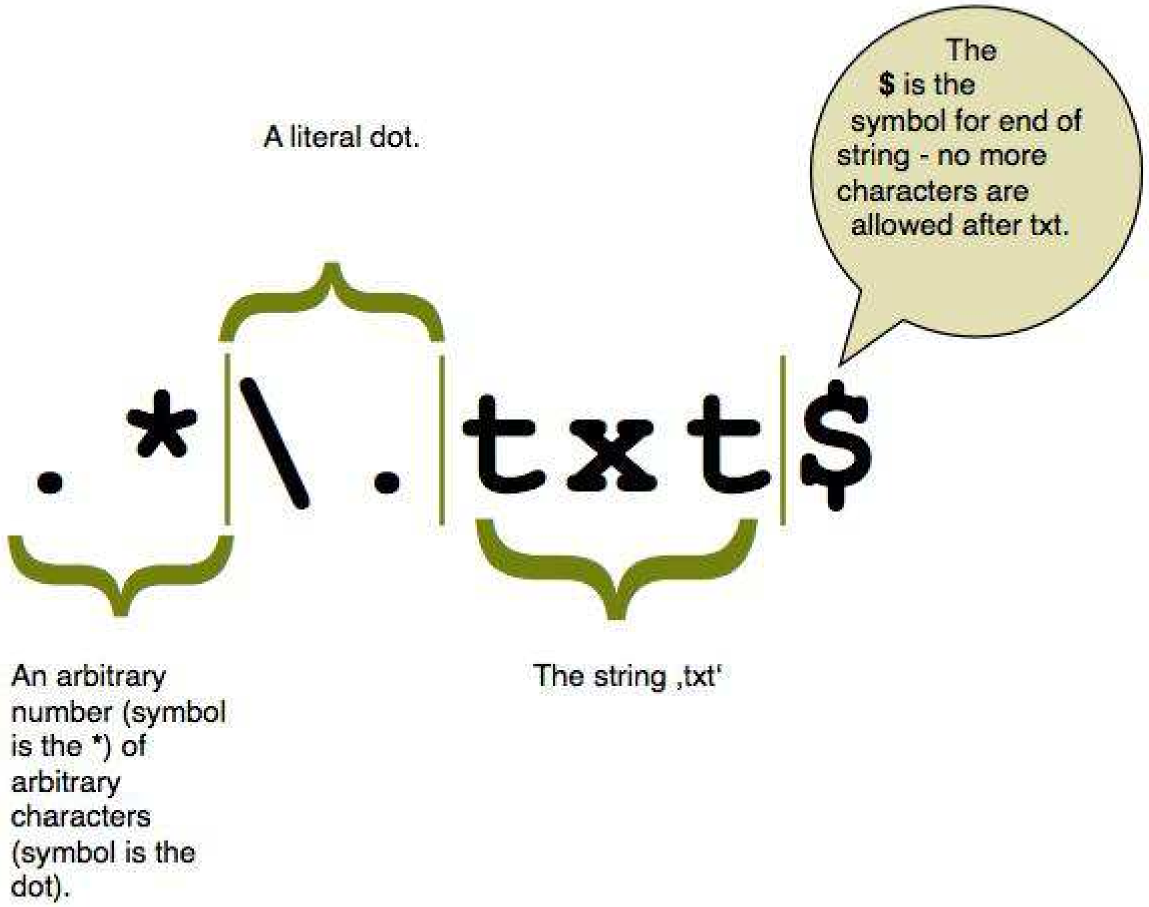

正则表达式的语法

一个正则表达式通常被称为一个模式（pattern），为用来描述或者匹配一系列匹配某个句法规则的字符串。例如：`Handel`、`Händel` 和 `Haendel` 这三个字符串，都可以由 `H(a|ä|ae)ndel` 这个模式来描述。

普通字符

普通字符包括没有显式指定为元字符的所有可打印和不可打印字符。这包括所有大写和小写字母、所有数字、所有标点符号和一些其他符号。

非打印字符

非打印字符也可以是正则表达式的组成部分。下表列出了表示非打印字符的转义序列：

字符	匹配	等效于
\f	换页符。	\x0c 和 \cL
\n	换行符。	\x0a 和 \cJ
\r	回车符。	\x0d 和 \cM
\s	任何空白字符。 其中包括空格、制表符和换页符。	[\f\n\r\t\v]
\S	任何非空白字符。	[^ \f\n\r\t\v]
\t	Tab 字符。	\x09 和 \cI
\v	垂直制表符。	\x0b 和 \cK

特殊字符

下表包含了单字符元字符的列表以及它们在正则表达式中的行为。

注意：若要匹配这些特殊字符之一，必须首先转义字符，即，在字符前面加反斜杠字符 (\)。例如，若要搜索“+”文本字符，可使用表达式“\+”。

元字符	行为	示例
*	零次或多次匹配前面的字符或子表达式。<br><br>等效于 {0,}。	zo* 与 "z" 和 "zoo" 匹配。
+	一次或多次匹配前面的字符或子表达式。<br><br>等效于 {1,}。	zo+ 与 "zo" 和 "zoo" 匹配，但与 "z" 不匹配。
?	零次或一次匹配前面的字符或子表达式。<br><br>等效于 {0,1}。<br><br>当 ? 紧随任何其他限定符（*、+、?、{*n*}、{*n*,} 或 {*n*,*m*}）之后时，匹配模式是非贪婪的。 非贪婪模式匹配搜索到的、尽可能少的字符串， 而默认的贪婪模式匹配搜索到的、尽可能多的字符串。	zo? 与 "z" 和 "zo" 匹配，但与 "zoo" 不匹配。<br><br>o+? 只与 "oooo" 中的单个 "o" 匹配，而 o+ 与所有 "o" 匹配。<br><br>do(es)? 与 "do" 或 "does" 中的 "do" 匹配。
^	匹配搜索字符串开始的位置。 如果标志中包括 m（多行搜索）字符，^ 还将匹配 \n 或 \r 后面的位置。<br><br>如果将 ^ 用作括号表达式中的第一个字符，则会对字符集求反。	^\d{3} 与搜索字符串开始处的 3 个数字匹配。<br><br>[^abc] 与除 a、b 和 c 以外的任何字符匹配。
$	匹配搜索字符串结尾的位置。 如果标志中包括 m（多行搜索）字符，^ 还将匹配 \n 或 \r 前面的位置。	\d{3}$ 与搜索字符串结尾处的 3 个数字匹配。
.	匹配除换行符 \n 之外的任何单个字符。 若要匹配包括 \n 在内的任意字符，请使用诸如 [\s\S] 之类的模式。	a.c 与 "abc" 、 "a1c" 和 "a-c" 匹配。
[]	标记括号表达式的开始和结尾。	[1-4] 与 "1" 、 "2" 、 "3" 或 "4" 匹配。 [^aAeEiIoOuU] 与任何非元音字符匹配。
{}	标记限定符表达式的开始和结尾。	a{2,3} 与 "aa" 和 "aaa" 匹配。
()	标记子表达式的开始和结尾。 可以保存子表达式以备将来之用。	A(\d) 与 "A0" 至 "A9" 匹配。 保存该数字以备将来之用。
|	指示在两个或多个项之间进行选择。	z|food 与 "z" 或 "food" 匹配。 (z|f)ood 与 "zood" 或 "food" 匹配。
/	表示 JScript 中的文本正则表达式模式的开始或结尾。 在第二个 "/" 后添加单字符标志可以指定搜索行为。	/abc/gi 是与 "abc" 匹配的 JScript 文本正则表达式。 g（全局）标志指定查找模式的所有匹配项，i（忽略大小写）标志使搜索不区分大小写。
\	将下一字符标记为特殊字符、文本、反向引用或八进制转义符。	\n 与换行符匹配。 \(与 "(" 匹配。 \\ 与 "\" 匹配。

大多数特殊字符在括号表达式内出现时失去它们的意义，并表示普通字符。

元字符

元字符	行为	示例
\b	与一个字边界匹配；即字与空格间的位置。	er\b 与 "never" 中的 "er" 匹配，但与 "verb" 中的 "er" 不匹配。
\B	非边界字匹配。	er\B 与 "verb" 中的 "er" 匹配，但与 "never" 中的 "er" 不匹配。
\d	数字字符匹配。<br><br>等效于 [0-9]。	在搜索字符串 "12 345" 中，\d{2} 与 "12" 和 "34" 匹配。 \d 与 "1" 、 "2" 、 "3" 、 "4" 和 "5" 匹配。
\D	非数字字符匹配。	\D+ 与 "abc123 def" 中 [illegible]

<table>
<tr><td></td><td>等效于 [^0-9]。</td><td>的“abc”和“def”匹配。</td></tr>
<tr><td>\w</td><td>与以下任意字符匹配：A-Z、a-z、0-9 和下划线。

等效于 [A-Za-z0-9_]。</td><td>在搜索字符串“The quick brown fox…”中，\w+ 与“The”、“quick”、“brown”和“fox”匹配。</td></tr>
<tr><td>\W</td><td>与除 A-Z、a-z、0-9 和下划线以外的任意字符匹配。

等效于 [^A-Za-z0-9_]。</td><td>在搜索字符串“The quick brown fox…”中，\W+ 与“…”和所有空格匹配。</td></tr>
<tr><td>[xyz]</td><td>字符集。 与任何一个指定字符匹配。</td><td>[abc] 与“plain”中的“a”匹配。</td></tr>
<tr><td>[^xyz]</td><td>反向字符集。 与未指定的任何字符匹配。</td><td>[^abc] 与“plain”中的“p”、“l”、“i”和“n”匹配。</td></tr>
<tr><td>[a-z]</td><td>字符范围。 匹配指定范围内的任何字符。</td><td>[a-z] 与“a”到“z”范围内的任何小写字母字符匹配。</td></tr>
<tr><td>[^a-z]</td><td>反向字符范围。 与不在指定范围内的任何字符匹配。</td><td>[^a-z] 与不在范围“a”到“z”内的任何字符匹配。</td></tr>
<tr><td>{n}</td><td>正好匹配 n 次。 n 是非负整数。</td><td>o{2} 与“Bob”中的“o”不匹配，但与“food”中的两个“o”匹配。</td></tr>
<tr><td>{n,}</td><td>至少匹配 n 次。 n 是非负整数。

* 与 {0,} 相等。

+ 与 {1,} 相等。</td><td>o{2,} 与“Bob”中的“o”不匹配，但与“foooood”中的所有“o”匹配。</td></tr>
<tr><td>{n,m}</td><td>匹配至少 n 次，至多 m 次。 n 和 m 是非负整数，其中 n <= m。 逗号和数字之间不能有空格。

? 与 {0,1} 相等。</td><td>在搜索字符串“1234567”中，\d{1,3} 与“123”、“456”和“7”匹配。</td></tr>
<tr><td>(模式)</td><td>与模式匹配并保存匹配项。 您可以从由 JScript 中的 exec Method返回的数组元素中检索保存的匹配项。 若要匹配括号字符 ( )，请使用“\(”或者“\)”。</td><td>(Chapter|Section) [1-9] 与“Chapter 5”匹配，保存“Chapter”以备将来之用。</td></tr>
<tr><td>(?:模式)</td><td>与模式匹配，但不保存匹配项；即不会存储匹配项以备将来之用。 这对于用“or”字符 (|) 组合模式部件的情况很有用。</td><td>industr(?:y|ies) 与 industry|industries 相等。</td></tr>
<tr><td>(?=模式)</td><td>正预测先行。 找到一个匹配项后，将在匹配文本之前开始搜索下一个匹配项。 不会保存匹配项以备将来之用。</td><td>^(?=.*\d).{4,8}$ 对密码应用以下限制：其长度必须介于 4 到 8 个字符之间，并且必须至少包含一个数字。

在该模式中，.*\d 查找后跟有数字的任意多个字符。 对于搜索字符串“abc3qr”，这与“abc3”匹配。

从该匹配项之前（而不是之后）开始，.{4,8} 与包含 4-8 个字符的字符串匹配。 这与“abc3qr”匹配。

^ 和 $ 指定搜索字符串的开始和结束位置。 这将在搜索字符串包含匹配字符之外的任何字符时阻止匹配。</td></tr>
<tr><td>(?!模式)</td><td>负预测先行。 匹配与模式不匹配的搜索字符串。 找到一个匹配项后，将在匹配文本之前开始搜索下一个匹配项。 不会保存匹配项以备将来之用。</td><td>\b(?!th)\w+\b 与不以“th”开头的单词匹配。

在该模式中，\b 与一个字边界匹配。 对于搜索字符串“ quick ”，这与第一个空格匹配。 (?!th) 与非“th”字符串匹配。 这与“qu”匹配。

从该匹配项开始，\w+ 与一个字匹配。 这与“quick”匹配。</td></tr>
<tr><td>\cx</td><td>匹配 x 指示的控制字符。 x 的值必须在 A-Z 或 a-z 范围内。 如果不是这样，则假定 c 就是文本“c”字符本身。</td><td>\cM 与 Ctrl+M 或一个回车符匹配。</td></tr>
<tr><td>[illegible]</td><td>[illegible]</td><td>[illegible]</td></tr>
</table>

\x*n*	匹配 *n*，此处的 *n* 是一个十六进制转义码。 十六进制转义码必须正好是两位数长。 允许在正则表达式中使用 ASCII 代码。	\x41 与 "A" 匹配。 \x041 等效于后跟有 "1" 的 "\x04" （因为 *n* 必须正好是两位数）。
num	匹配 *num*，此处的 *num* 是一个正整数。 这是对已保存的匹配项的引用。	(.)\1 与两个连续的相同字符匹配。
n	标识一个八进制转义码或反向引用。 如果 *n* 前面至少有 *n* 个捕获子表达式，那么 *n* 是反向引用。 否则，如果 *n* 是八进制数 (0-7)，那么 *n* 是八进制转义码。	(\d)\1 与两个连续的相同数字匹配。
nm	标识一个八进制转义码或反向引用。 如果 *nm* 前面至少有 *nm* 个捕获子表达式，那么 *nm* 是反向引用。 如果 *nm* 前面至少有 *n* 个捕获子表达式，则 *n* 是反向引用，后面跟有文本 *m*。 如果上述情况都不存在，当 *n* 和 *m* 是八进制数字 (0-7) 时，*nm* 匹配八进制转义码 *nm*。	\11 与制表符匹配。
nml	当 *n* 是八进制数字 (0-3)，*m* 和 *l* 是八进制数字 (0-7) 时，匹配八进制转义码 *nml*。	\011 与制表符匹配。
\u*n*	匹配 *n*，其中 *n* 是以四位十六进制数表示的 Unicode 字符。	\u00A9 与版权符号 (©) 匹配。

顺序

正则表达式的计算方式与算术表达式非常类似；即从左到右进行计算，并遵循优先级顺序。

字符具有高于替换运算符的优先级，例如，允许“m|food”匹配“m”或“food”。

一些具体的例子

数字

```
数字：^[0-9]*$
n位的数字：^\d{n}$
至少n位的数字：^\d{n,}$
m-n位的数字：^\d{m,n}$
零和非零开头的数字：^(0|[1-9][0-9]*)$
非零开头的最多带两位小数的数字：^([1-9][0-9]*)+(.[0-9]{1,2})?$
带1-2位小数的正数或负数：^(\-)?\d+(\.\d{1,2})?$
正数、负数、和小数：^(\-|\+)?\d+(\.\d+)?$
有两位小数的正实数：^[0-9]+(.[0-9]{2})?$
有1~3位小数的正实数：^[0-9]+(.[0-9]{1,3})?$
非零的正整数：^[1-9]\d*$ 或 ^([1-9][0-9]*){1,3}$ 或 ^\+?[1-9][0-9]*$
非零的负整数：^\-[1-9][]0-9"*$ 或 ^-[1-9]\d*$
非负整数：^\d+$ 或 ^[1-9]\d*|0$
非正整数：^-[1-9]\d*|0$ 或 ^((-\d+)|(0+))$
非负浮点数：^\d+(\.\d+)?$ 或 ^[1-9]\d*\.\d*|0\.\d*[1-9]\d*|0?\.0+|0$
非正浮点数：^((-\d+(\.\d+)?)|(0+(\.0+)?))$ 或 ^(-([1-9]\d*\.\d*|0\.\d*[1-9]\d*))|0
正浮点数：^[1-9]\d*\.\d*|0\.\d*[1-9]\d*$ 或 ^(([0-9]+\.[0-9]*[1-9][0-9]*)|([0-9]*[
```

```
负浮点数：^-([1-9]\d*\.\d*|0\.\d*[1-9]\d*)$ 或 ^(-(([0-9]+\.[0-9]*[1-9][0-9]*)|([0
浮点数：^(-?\d+)(\.\d+)?$ 或 ^-?([1-9]\d*\.\d*|0\.\d*[1-9]\d*|0?\.0+|0)$
```

字符相关

```
汉字：^[\u4e00-\u9fa5]{0,}$
英文和数字：^[A-Za-z0-9]+$ 或 ^[A-Za-z0-9]{4,40}$
长度为3-20的所有字符：^.{3,20}$
由26个英文字母组成的字符串：^[A-Za-z]+$
由26个大写英文字母组成的字符串：^[A-Z]+$
由26个小写英文字母组成的字符串：^[a-z]+$
由数字和26个英文字母组成的字符串：^[A-Za-z0-9]+$
由数字、26个英文字母或者下划线组成的字符串：^\w+$ 或 ^\w{3,20}$
中文、英文、数字包括下划线：^[\u4E00-\u9FA5A-Za-z0-9_]+$
中文、英文、数字但不包括下划线等符号：^[\u4E00-\u9FA5A-Za-z0-9]+$ 或 ^[\u4E00-\u9FA5A-Z
可以输入含有^%&',;=?$\"等字符：[^%&',;=?$\x22]+
禁止输入含有~的字符：[^~\x22]+
```

特殊

```
Email地址：^\w+([-+.]\w+)*@\w+([-.]\w+)*\.\w+([-.]\w+)*$
域名：[a-zA-Z0-9][-a-zA-Z0-9]{0,62}(/.[a-zA-Z0-9][-a-zA-Z0-9]{0,62})+/.?
InternetURL：[a-zA-z]+://[^\s]* 或 ^http://([\w-]+\.)+[\w-]+(/[\w-./?%&=]*)?$
手机号码：^(13[0-9]|14[5|7]|15[0|1|2|3|5|6|7|8|9]|18[0|1|2|3|5|6|7|8|9])\d{8}$
电话号码("XXX-XXXXXXX"、"XXXX-XXXXXXXX"、"XXX-XXXXXXX"、"XXX-XXXXXXXX"、"XXXXXXX"和
国内电话号码(0511-4405222、021-87888822)：\d{3}-\d{8}|\d{4}-\d{7}
身份证号(15位、18位数字)：^\d{15}|\d{18}$
短身份证号码(数字、字母x结尾)：^([0-9]){7,18}(x|X)?$ 或 ^\d{8,18}|[0-9x]{8,18}|[0-9X]
帐号是否合法(字母开头，允许5-16字节，允许字母数字下划线)：^[a-zA-Z][a-zA-Z0-9_]{4,15}$
密码(以字母开头，长度在6~18之间，只能包含字母、数字和下划线)：^[a-zA-Z]\w{5,17}$
强密码(必须包含大小写字母和数字的组合，不能使用特殊字符，长度在8-10之间)：^(?=.*\d)(?=.*[a-
日期格式：^\d{4}-\d{1,2}-\d{1,2}
一年的12个月(01～09和1～12)：^(0?[1-9]|1[0-2])$
一个月的31天(01～09和1～31)：^((0?[1-9])|((1|2)[0-9])|30|31)$
```

我们的问题

有了上面的学习经验，我们的问题其实很容易解决。我们需要做的是把他们替换成空字符，或类似的替换。

tag问题

```
"</?.+?>"
```

数字问题

```
"\d+\n\d{2}:\d{2}:\d{2},\d{3} --> \d{2}:\d{2}:\d{2},\d{3}"
```

语言选择

Clojure介绍

Clojure是Lisp编程语言在Java平台上的现代、动态及函数式方言。与其他Lisp一样，Clojure视代码为数据且拥有一套Lisp宏系统。 其开发过程目前由社区驱动。Clojure提倡不可变性（immutability）与持久数据结构（persistent data structures）并鼓励程序员显式地管理标识（identity）及其状态（state）。对利用不可变值（immutable values）及显式时间进展构造（explicit progression-of-time constructs）进行编程的专注旨在促进更加健壮的（尤其是并发）程序的开发。Clojure的类型系统是完全动态的，但人们近期也开始探索其基于渐进类型化（gradual typing）的实现。

Clojure有什么优点

Clojure能够吸引人的很重要一点是它是JVM之上的语言，这个决定非常关键。

首先，因为根植于JVM之上，并且做到了跟Java语言的相互调用，它能吸引很多成熟的Java开发者。其次，它可以使用Java社区丰富的开源软件，不需要从头去构建一个社区，你可以看到很多Clojure开源代码都是简单地包装Java的开源包，但是通过Clojure高度抽象简单的语法提供更便利的使用的方式； 第三，由于JVM平台本身的高度成熟和优化，clojure的编译器生成

的byte code跟Java编译器生成的byte code并无二致（不完全是），它的性能和稳定性也能马上得到保证，这比从头构建一个新平台成本低得多。

构建于JVM之上，Clojure就是一门“严肃”的语言，而非很多人眼中的Lisp“玩具”语言，你学习后可以马上使用并且实践。但是Clojure又是Lisp方言，Lisp的神奇能力它还都保留，这样兼具美学和实用的语言如何让人不爱？我相信很多熟悉Scheme之类方言的童鞋，并且有Java背景的，都会对Clojure有相见

恨晚的感觉。

Clojure的设计原则可以概括成5个词汇：简单、专注、实用、一致和清晰

1. 简单： 鼓励纯函数，极简的语法（少数special form），个人也认为clojure不能算是多范式的语言（有部分OO特性），为了支持多范式引入的复杂度，我们在C++和Scala身上都看到了。
2. 专注：前缀运算符不需要去考虑优先级，也没有什么菱形继承的问题，动态类型系统（有利有弊），REPL提供的探索式编程方法（告别修改/编译/运行的死循环，所见即所得）。
3. 实用：前面提到，构建在JVM之上，跟Java语言的互操作非常容易。直接调用Java方法，不去发明一套新的调用语法，努力规避Java语言中繁琐的地方(doto,箭头宏等等）。
4. 清晰：纯函数（前面提到），immutable var，immutable数据结构，STM避免锁问题。不可变减少了心智的负担，降低了多线程编程的难度，纯函数也更利于测试和调试。
5. 一致：语法的一致性：例如doseq和for宏类似，都支持destructring,支持相同的guard语句（when,while）。数据结构的一致性：sequence抽象之上的各种高阶函数。

Hello World

```
(defn hello ; 函数名
  [& args] ; 参数向量 (`&`表示可变参数)
  (println "Hello, World!")) ; 函数体

(hello)
```

Clojure环境搭建

我们使用Clojure官方的deps.edn来配置我们的环境，这里只做了两件事，第一件是选择了目前最新的版本的Clojure作为我们的工作语言。第二件是把我们将会使用的包加入到我们的工作环境中。非常简单，因为NextJournal的强大，一切都如此美妙。

```
{:deps
 {org.clojure/clojure {:mvn/version "1.10.1"}
  compliment {:mvn/version "0.3.9"}
  clojure-opennlp/clojure-opennlp {:mvn/version "RELEASE"}
  com.kennycason/kumo-core {:mvn/version "RELEASE"}
  com.hypirion/clj-xchart {:mvn/version "RELEASE"}}}
```

我们来看看版本对不对。

```
{:hello (clojure-version)} ;; see which of clojure it is running
```

看起来是可以的。那我们就准备开始出发了。

代码实现

语料库生成

这里我们的任务包括如下两点:

- 首先需要从目录里读取文件，并对每一个文件操作，最后生成一个语料库文件。
- 对每个文件而言，需要完成上述的两点替换工作。
- 因为会经常执行程序，所以一定要保证之前生成的语料库在每次操作之前，都被删除。

```
(require '[clojure.string :as string])
(require '[clojure.java.io :as io])

(defn rm
  "delete existing corpus, and create a new one afterwards"
  [& _]
  (io/delete-file "subtitles_corpus.dat" true))

(defn mk
  "clean subtitles and make it a tiny text corpus"
  [& _]
```

```
  (let [fs (next (file-seq (io/file "/subtitles/srt")))
        ;; using regexp to clean the subtitles
        ;; time-stamp and scene numbers
        ts #"\d+\n\d{2}:\d{2}:\d{2},\d{3} --> \d{2}:\d{2}:\d{2},\d{3}"
        ;; html tags, for italics and bolds, etc
        ht #"</?.+?>"
        ;; empty lines
        el #"\n+"
        ;; functions to replace them
        ts! #(string/replace % ts "")
        ht! #(string/replace % ht "")
        el! #(string/replace % el "\n")]
    (for [f fs
          ;; slurp it and then clean it one by one
          :let [c (->> (slurp (str f))
                       (ts!)
                       (ht!)
                       (el!))]]
      (spit "/subtitles/subtitles_corpus.dat" c
            ;; append to ensure it will be a single file with all the contents
            :append true))))

(rm)
(mk)
```

下面我们看看到底生成文件没有。

```
ls /subtitles
```

果然已经生成了文件，那么我们就来看看怎么样吧。

```
bat -r :200 /subtitles/subtitles_corpus.dat
```

不错，正是我们想要的。我们现在就可以把这个文件用我们常用的语料库分析软件进行分析和使用了。这里我们选择Antconc。

把语料库变成一朵云

Antconc虽然简明扼要，功能实用，但是抽象的数字结果往往不能满足用户的需要，我们考虑使用Word Cloud技术，将生成的语料变成鲜明的词云，通过云朵的大小来反映在语料库中，具体词汇出现的频率大小。从感官上来讲，具有更强的冲击力，也具有更好的效果。

```clojure
(import [java.awt Dimension Color])
(import [com.kennycason.kumo WordCloud CollisionMode])
(import [com.kennycason.kumo.bg CircleBackground])
(import [com.kennycason.kumo.palette LinearGradientColorPalette])
(import [com.kennycason.kumo.font.scale SqrtFontScalar])
(import [com.kennycason.kumo.nlp FrequencyAnalyzer])

(defn wc
  "function for word cloud"
  [& _]
  (let [fa (FrequencyAnalyzer.)
        _ (.setWordFrequenciesToReturn fa 600)
        fq (.load fa "subtitles_corpus.dat")
        d (Dimension. 1300 1300)
        sqfs(SqrtFontScalar. 25 90)
        lgcp (LinearGradientColorPalette. Color/MAGENTA
                                          Color/CYAN Color/DARK_GRAY 80 80)
        cbg (CircleBackground. 650)
        wc (WordCloud. d CollisionMode/PIXEL_PERFECT)]
    (doto wc
      (.setPadding 2)
      (.setColorPalette lgcp)
      (.setBackground cbg)
      (.setFontScalar sqfs)
      (.build fq)
      (.writeToFile "subtitles_corpus.png"))))

(wc)
```

Clojure在NextJournal里没有办法直接把图像显示出来，我们这里选择把生成的图上传到网上，再来看。

```
curl -F 'name=@/subtitles/subtitles_corpus.png' https://img.vim-cn.com/
```

不过我也上传了一份目前的图方便大家直接观看。

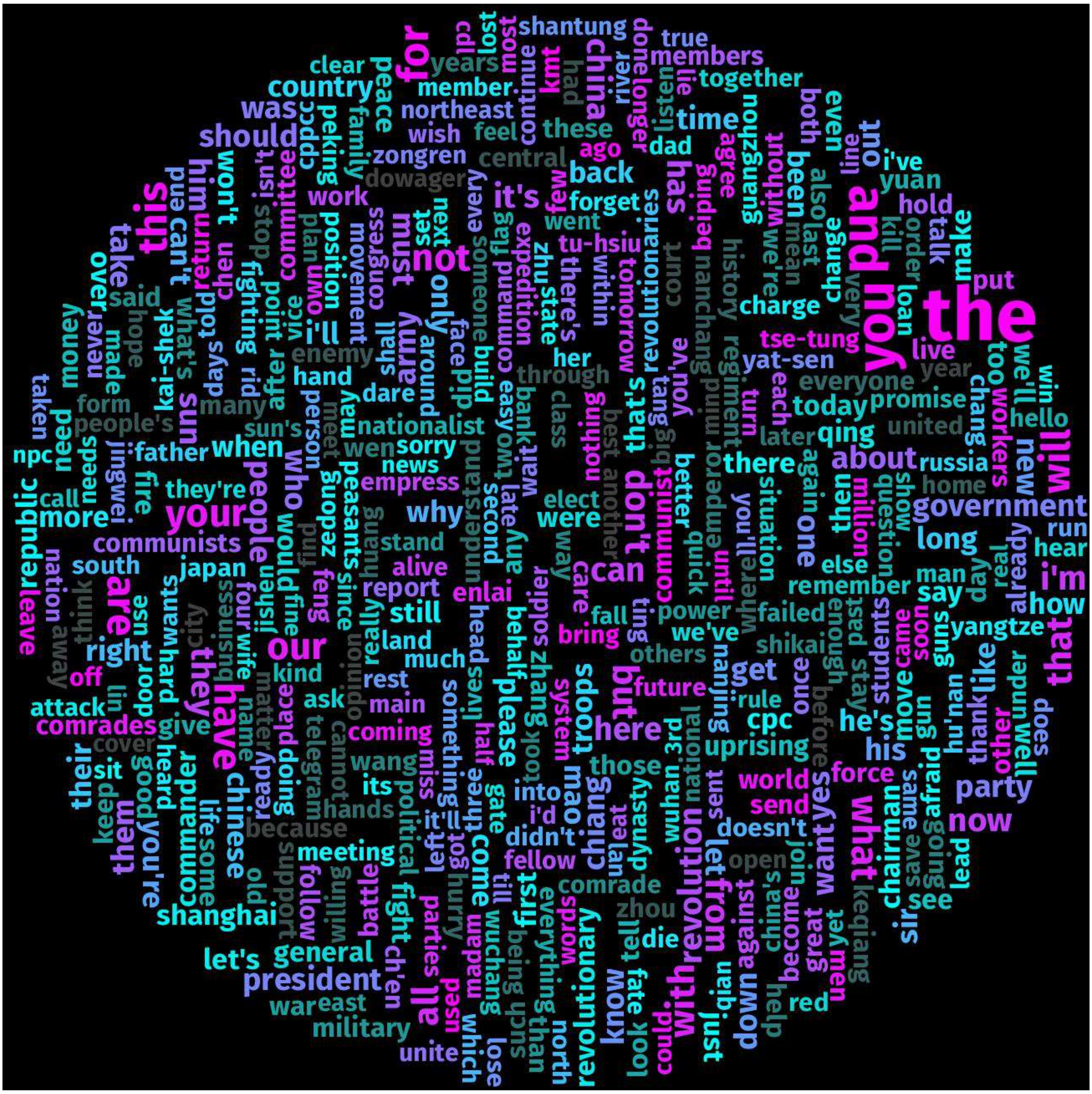

看起来虽然比较粗糙，但是已经是一个成型的词云了。未来，我们还会对美观和一些常见词的排除进行优化。但是这不是目前的重点。

看看语料的语言学属性

上面两个步骤，我们生成了我们的迷你语料库，用Antconc对它进行分析，同时也使用小程序生成了词云，更好的显示效果。现在我们来对语料库文本进行一些最基本的语言学分析和统计学分析。

在这里，我们希望对文本进行完整的词性标注。其次，将词性标注的结果绘制成为饼状图。我们这里使用了宾夕法尼亚大学的词性标注方式。

```
(require '[opennlp.nlp :refer [make-pos-tagger make-tokenizer]])
(require '[com.hypirion.clj-xchart :as c])
(require '[clojure.pprint :refer [pprint]])


(defn pos
  "use opennlp to show pos taggings"
  [& _]
  (let [pos-tag (make-pos-tagger "models/en-pos-maxent.bin")
        tokenize (make-tokenizer "models/en-token.bin")
        s (slurp "subtitles_corpus.dat")
        pos (pos-tag (tokenize s))
        fq (take-last 10 (sort-by val (frequencies (vals (into (sorted-map) pos)
    (pprint pos)
    (pprint fq)
    (c/spit (c/pie-chart fq {:title "POS Pie Chart" :render-style :donut :theme

(pos)
```

如上就是分析的结果，看起来很多很多，我们将其绘制成为了饼状图。因为NextJournal的虚拟环境不包括X11的图形界面，所以我们使用Java Swing生成的图形没有办法直接显示。这里选择保存为PNG图片格式。我们依然首先上传到网络。

```
ls /subtitles
```

```
curl -F 'name=@/subtitles/pos.png' https://img.vim-cn.com/
```

同时也保存了一份目前的状态。供大家参考。

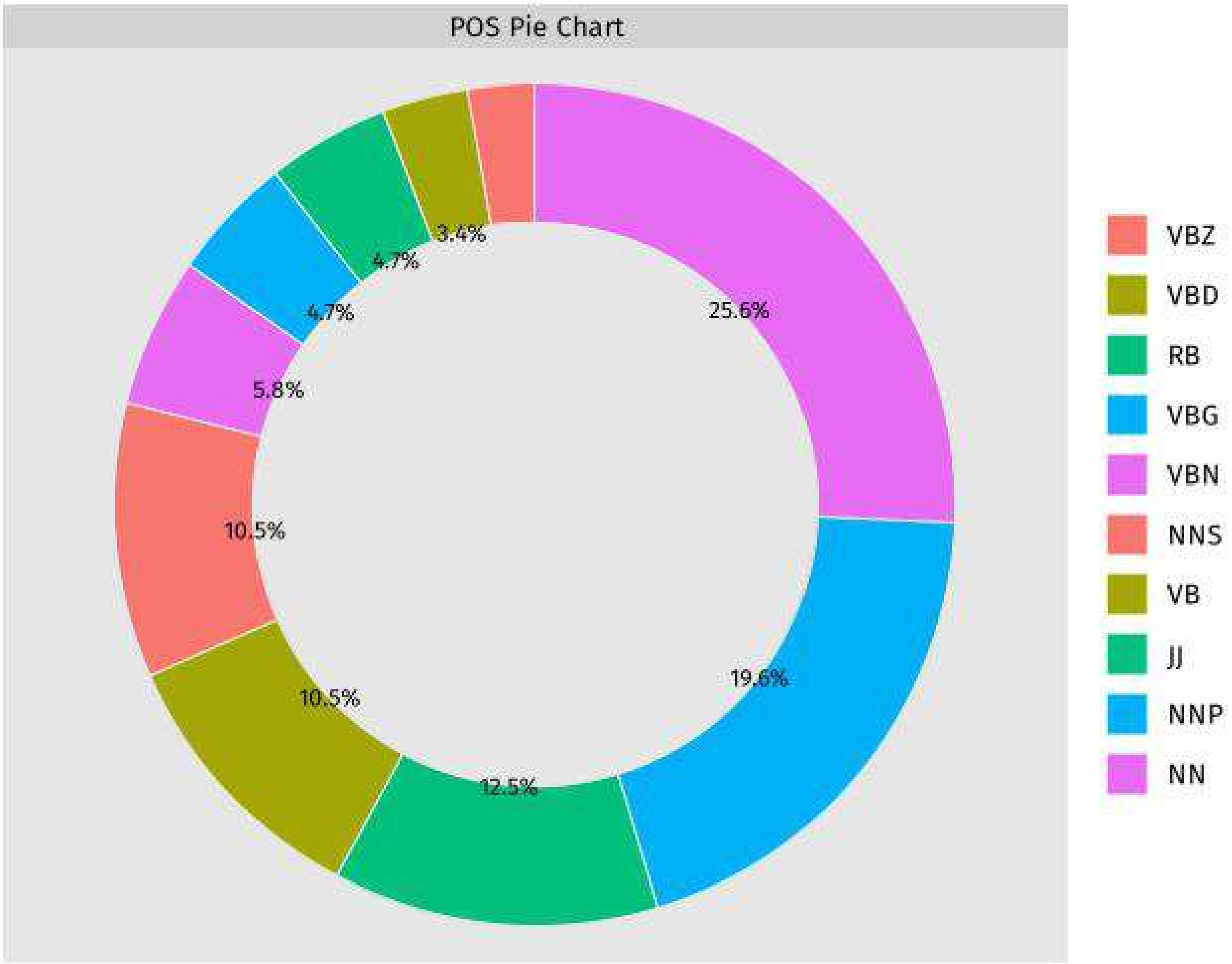

结束语

为了这个短暂的实验，我们花了三四天的时间去思考，花了一天多的时间去实现。尽管只是一个baby project和一个toy corpus，但是我们为我们的计划设定了非常宏大的目标和长远的规划。

当然，我们项目组的两位成员都还处在学习的早期阶段，我们特别希望，作为我们的第一个小项目，这是我们奋斗的起点，更是时刻鞭策我们前进的动力源泉。希望在不久的将来，我们可以把这个baby project做的更大更强。

非常感谢您的时间。也希望您多提宝贵的意见。

> When my dream was near the moon,
>
> The white folds of its gown
>
> Filled with yellow light.

> The soles of its feet
>
> Grew red.
>
> Its hair filled
>
> With certain blue crystallizations
>
> From stars,
>
> Not far off.

未来的路还很长，我们希望我们可以一起仰望满天的繁星。✨

This notebook was exported from
