# Supplementary material for: Teaching data science to undergraduate translation trainees: Pilot evaluation of a task-based course
Source: Front Psychol. 2022 Aug 3;13:939689. doi: 10.3389/fpsyg.2022.939689 (PMC9381704; doi:10.3389/fpsyg.2022.939689)
Supplement: Supplementary file 2 [file Data_Sheet_2.docx]

Appendix 2 – Students’ Learning Artifacts

1. Toy Chinese-English Translator Program


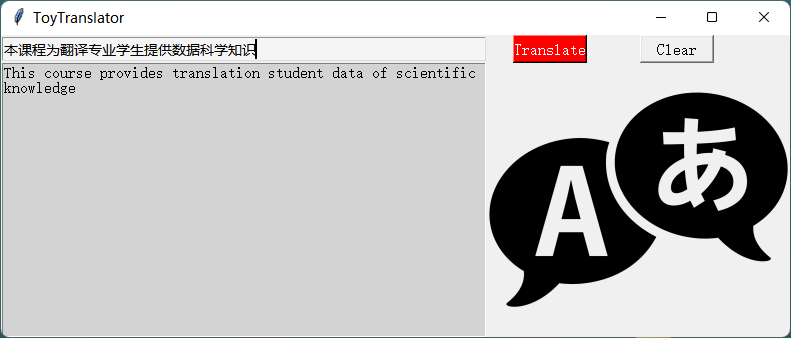


1. Mini website built with scraped data

| 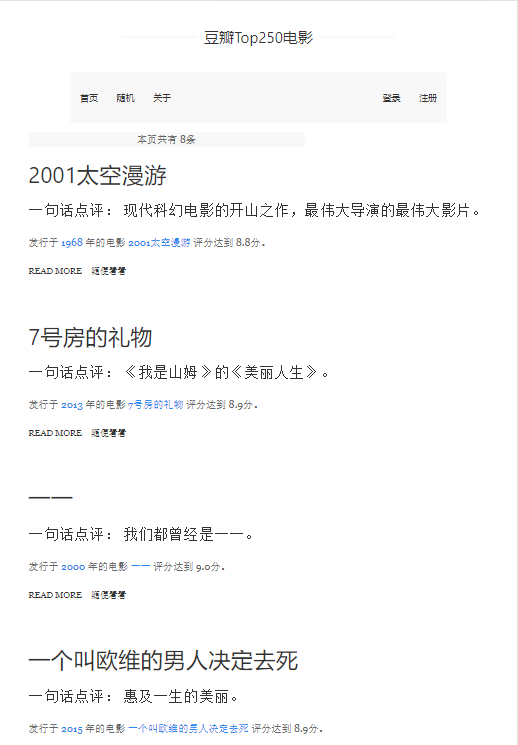 | 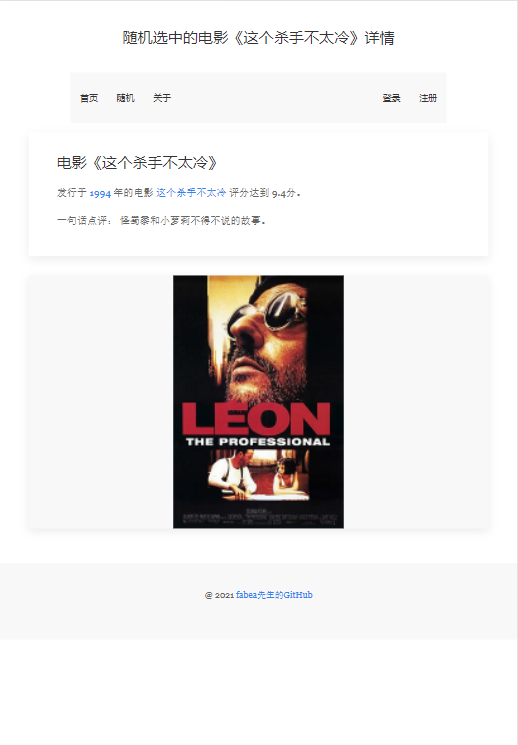 |
| --- | --- |
| 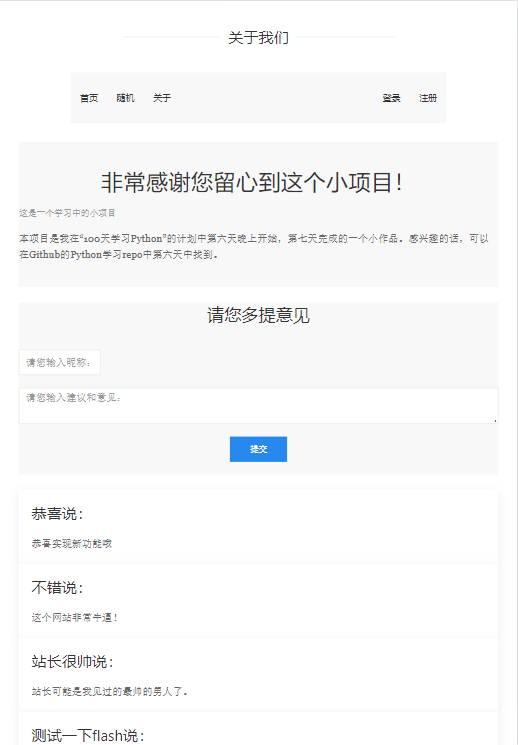 | 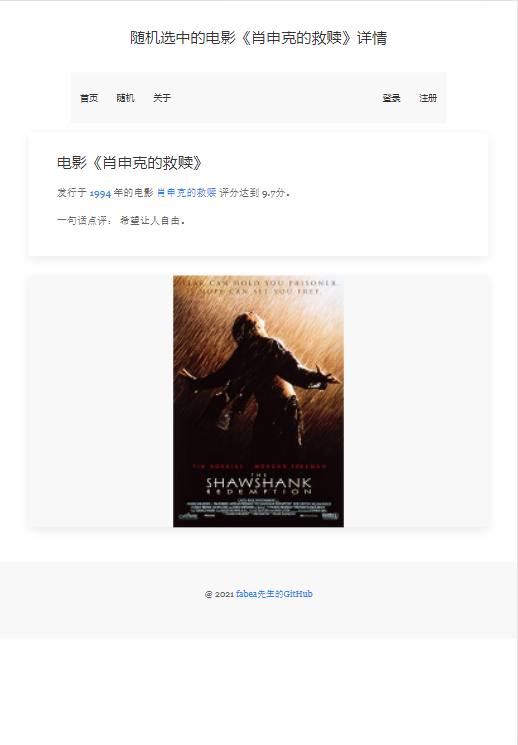 |

1. Student’s Thesis Proposal on Corpus Analysis of Subtitle Translation

| 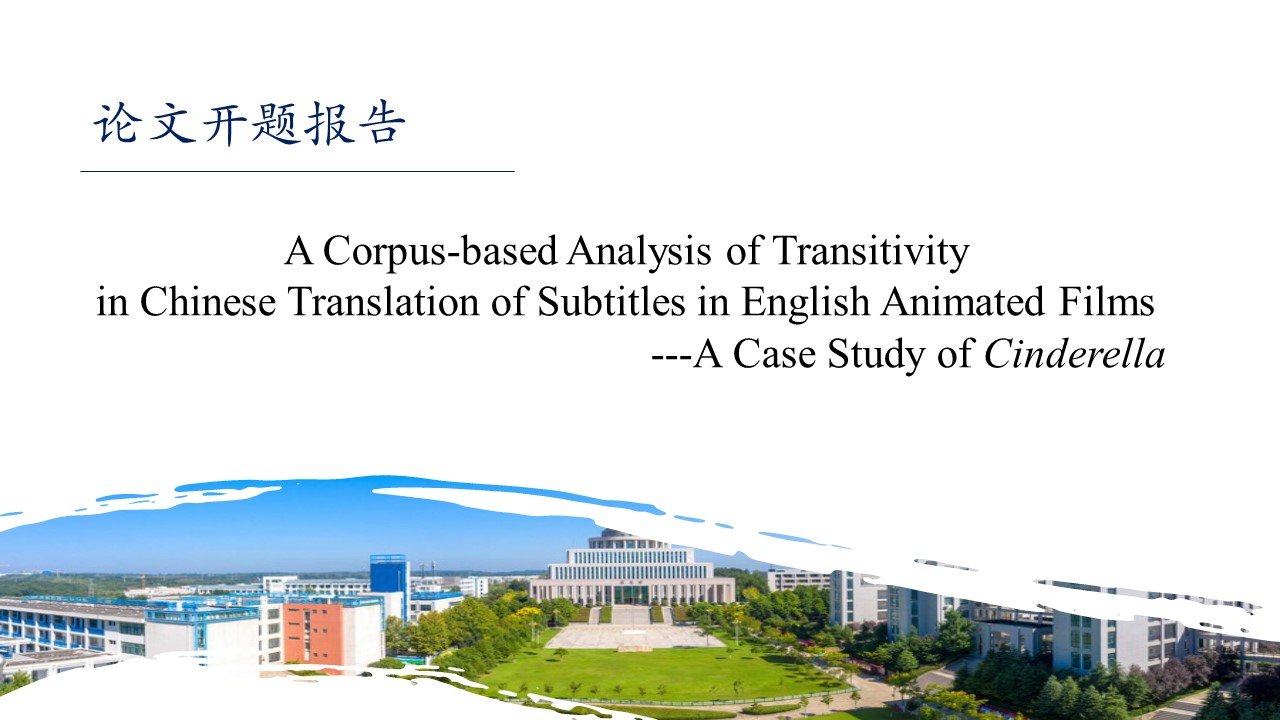 |
| --- |
| 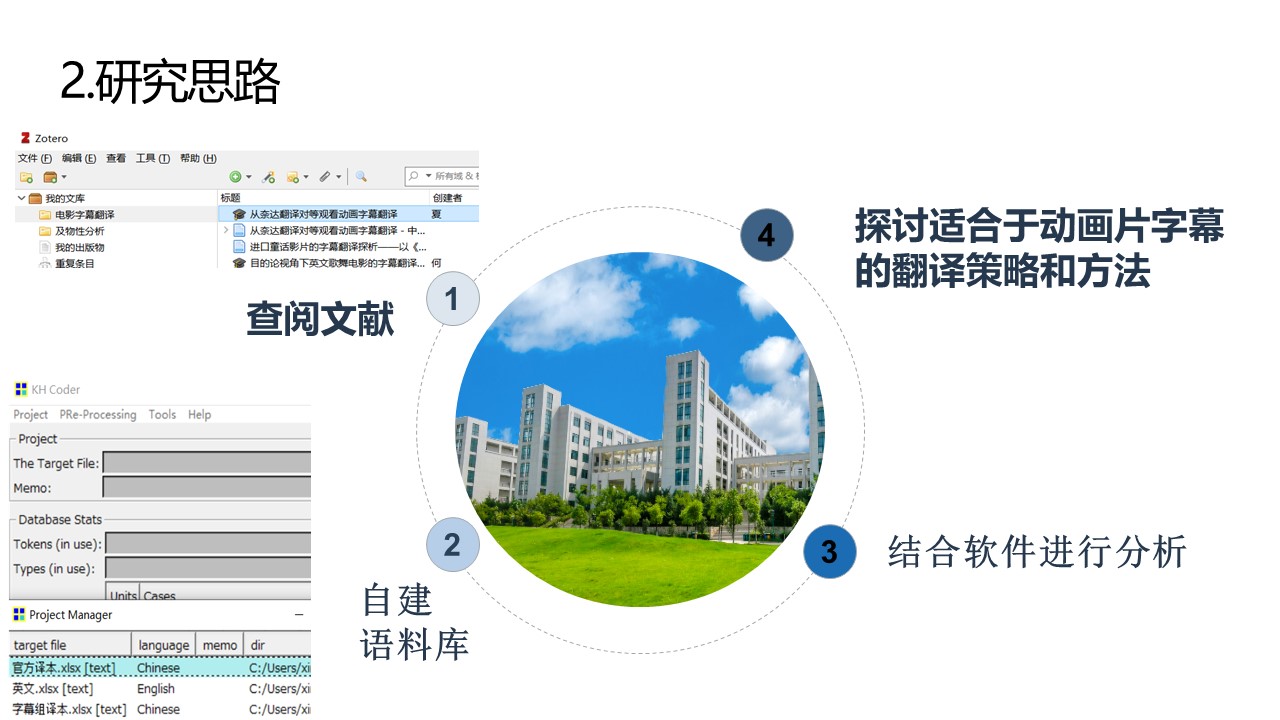 |
| 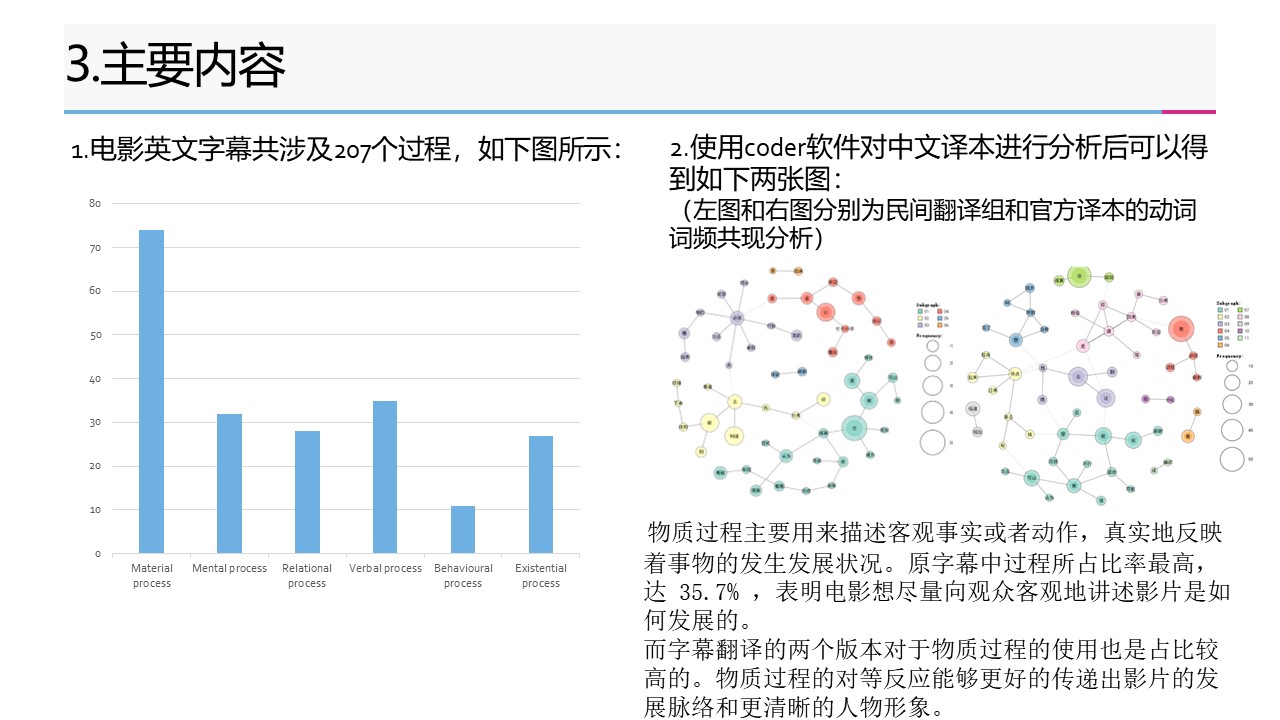 |
